# Clustering and Differential Alignment Algorithm: Identification of Early Stage Regulators in the *Arabidopsis thaliana* Iron Deficiency Response

**DOI:** 10.1371/journal.pone.0136591

**Published:** 2015-08-28

**Authors:** Alexandr Koryachko, Anna Matthiadis, Durreshahwar Muhammad, Jessica Foret, Siobhan M. Brady, Joel J. Ducoste, James Tuck, Terri A. Long, Cranos Williams

**Affiliations:** 1 Electrical and Computer Engineering, North Carolina State University, Raleigh, North Carolina, United States of America; 2 Plant and Microbial Biology, North Carolina State University, Raleigh, North Carolina, United States of America; 3 Plant Biology, University of California Davis, Davis, California, United States of America; 4 Civil, Construction, and Environmental Engineering, North Carolina State University, Raleigh, North Carolina, United States of America; Lady Davis Institute for Medical Research/McGill University, CANADA

## Abstract

Time course transcriptome datasets are commonly used to predict key gene regulators associated with stress responses and to explore gene functionality. Techniques developed to extract causal relationships between genes from high throughput time course expression data are limited by low signal levels coupled with noise and sparseness in time points. We deal with these limitations by proposing the Cluster and Differential Alignment Algorithm (CDAA). This algorithm was designed to process transcriptome data by first grouping genes based on stages of activity and then using similarities in gene expression to predict influential connections between individual genes. Regulatory relationships are assigned based on pairwise alignment scores generated using the expression patterns of two genes and some inferred delay between the regulator and the observed activity of the target. We applied the CDAA to an iron deficiency time course microarray dataset to identify regulators that influence 7 target transcription factors known to participate in the *Arabidopsis thaliana* iron deficiency response. The algorithm predicted that 7 regulators previously unlinked to iron homeostasis influence the expression of these known transcription factors. We validated over half of predicted influential relationships using qRT-PCR expression analysis in mutant backgrounds. One predicted regulator-target relationship was shown to be a direct binding interaction according to yeast one-hybrid (Y1H) analysis. These results serve as a proof of concept emphasizing the utility of the CDAA for identifying unknown or missing nodes in regulatory cascades, providing the fundamental knowledge needed for constructing predictive gene regulatory networks. We propose that this tool can be used successfully for similar time course datasets to extract additional information and infer reliable regulatory connections for individual genes.

## Introduction

Transcriptome studies are commonly used to assess differential gene activity. Differentially expressed genes identified as having DNA binding activity, termed Transcription Factors (TFs), are of interest due to their ability to control the activation and repression of gene expression, directly influencing the accumulation of RNA and proteins that control growth and stress responses. Given the importance of transcription factors in plant stress responses, development, and cell differentiation [1], the identification of key plant transcriptional regulators and their targets continues to be an area of intense research. Though many high throughput time course transcriptomic datasets are available, the prediction of regulator-target relationships between individual genes from these datasets remains an on-going area of research.

Much of what has been inferred from time course transcriptomic analysis regarding transcription factor involvement in stress responses comes from visual assessment of gene expression behavior followed by mutant screens [2–6]. These techniques are limited at inferring regulatory relationships between genes. Moreover, mutant screens in the absence of specific predictions can be time consuming and genes without mutant phenotypes are often disregarded. This lack of mutant phenotypes is because the combinatorial and often redundant function of a gene in a pathway results in the absence of a dramatic phenotype, making experimental identification and verification difficult. Computational inference approaches can increase our understanding of transcription factor involvement in stress response by creating testable hypotheses concerning regulatory relationships, revealing networks of interactions that could be easily missed when using mutant screens. Many regulatory network inference algorithms that use gene expression data start with a refined set of genes to generate predictions. These algorithms, therefore, can require extensive prior knowledge and are most appropriate for inferring structure [7–9] and/or mathematical relationships [10–12] based on a subset of genes consisting of known major players in the response. There remains a need for further development of computational algorithms that are able to predict gene regulatory relationships based on a full transcriptomic dataset with little prior knowledge.

We sought to develop such a computational approach to identify key regulator-target relationships involved in the iron deficiency stress response in *Arabidopsis thaliana*. Iron deficiency is a useful stress to help develop and test such an algorithm because: (1) iron homeostasis is tightly regulated by transcription factors [13]; (2) a previously published iron deficiency time course microarray data in *A. thaliana* roots was available [2, 4]; and (3) several transcription factors involved in iron deficiency homeostasis have been characterized and understanding the regulation of these transcription factors would be valuable to assist in the development of future applications in agriculture.

Previous iron deficiency studies have led to the identification of several key iron homeostasis transcription factors including *bHLH39* [14], *bHLH101* [15], *bHLH115* [4], *PYE* [4], *MYB10* [6], *MYB72* [6], and *BTS* [4, 16, 17]. These genes have altered expression after 12 hours of exposure to iron deficient conditions [4]. Little is known about transcription factors that are active before 12 hours or about how early regulators target or influence the expression of known iron homeostasis transcription factors. We focused on formulating and implementing a computational approach that can be applied to the iron deprivation dataset in Dinneny et al. [2] as well as other typical transcriptome time course datasets (microarray or RNA-Seq) to identify unknown regulator-target relationships under a series of challenges (e.g. missing prior information) that are common to other stress analyses. Given that more than 80% of biological time course stress datasets in *A. thaliana* include less than 8 (typically unevenly spaced) time points [18] and 3 or less replicates [2, 19, 20], we focused on addressing the identification of relationships in low resolution, unevenly sampled, and noisy time course data. We focused on formulating an algorithm that can work on as few as 4 time points. Effectiveness of the algorithm would in all likelihood increase with additional time resolution, particularly depending on the timing of the biological process of interest and sampling point selection with respect to this process. We also wanted to create an algorithm whose output is in the form of regulator-target connections between individual genes. An algorithm of this type would identify players involved in a transcriptional response cascade. With these players known and validated, further computational tools can be used to create more complex and predictive gene regulatory networks that capture the response of corresponding biological processing over time and that can be used to make predictions on various experimental scenarios [21]. A critical aspect of this is that the algorithm should result in a manageable set of putative candidates that can be experimentally validated. We emphasize here that in the case of the iron deficiency response, very few genetic players have been identified. This lack of knowledge prevents the accurate development of a dynamic gene regulatory network of the iron deficiency response. It is the case for this and many other stresses that identification of these initial set of players and relationships is a fundamental step toward the dynamic modeling and further analysis of these responses.

Although several gene regulatory connection inference algorithms exist in the literature [7, 22–24], the characteristics of the iron deprivation dataset and insufficient prior knowledge about interactions between iron response regulators present unique challenges that must be addressed. Gene regulatory network inference algorithms presented in the literature are shown in Table 1; none of which fully address the challenges associated with iron response analysis. Some algorithms require expression data from a limited set of genes [7–9] where others use expression data from evenly spaced time course experiments [24–26]. Some algorithms do not resolve regulator-target interactions between individual genes and focus more on broad relationships between clusters of genes [22]. In particular, a recent time-course based computational approach presented in Windram et al. [22] looked at formulating regulatory connections between plant transcription factor families in response to pathogen infection. By analyzing 24 equally spaced transcriptome samples under stressed and unstressed conditions, the authors were able to infer connections between clusters of genes that responded at different time stages. Using this approach to extract specific regulators that influence known iron homeostasis transcription factors would be challenging since inter-cluster connections do not imply relationships between all genes from the connected clusters [27]. Other algorithms that extract causal influences between pairs of genes, such as the Event Method algorithm in Kwon et al. [24], can be modified to analyze general datasets with uneven time course measurements. However, these algorithms can result in an extensive number of pairwise predictions. The application of a modified Event Method algorithm to the iron deprivation dataset yielded results that were unable to resolve the roles (regulator/target) for a significant number of individual gene pairs. Moreover, most connections that we found and experimentally validated were not resolved by the modified algorithm, as detailed in the Results section. Other algorithms require multiple transcriptome datasets [28, 29] or predict connections between genes based on correlation [30, 31], which without modification ignore temporal evidence provided by the type of dataset [24] and are likely to result in the prediction of coexpressed genes rather than regulator-target relationships.

**Table 1:** Regulatory interactions inference algorithms.

**Paper**	**Algorithm Capabilities**			
	Whole genome analysis	Uneven time course	Causality inference	Pairwise connections
Windram et al. [22]	✔		✔	
Nie et al. [23]	✔	✔		✔
Kwon et al. [24]	✔		✔	✔
Bickel et al. [25]				
Schmitt et al. [26]				
Barker et al. [7]		✔	✔	✔
Zhao et al. [8]				
Misra et al. [9]				

We developed the Cluster and Differential Alignment Algorithm (CDAA) to address the unique challenges associated with better understanding regulator-target interactions in the iron deprivation stress response. Key aspects of the algorithm include co-expression analysis [32] to associate each gene with a stage in the response process, relevance network inference techniques [33] to identify causal relationships between genes, and thresholding [18] to mitigate the effects of noise in the data. The algorithm groups genes showing transcriptional activity at different time intervals into stages and looks for similarities in expression behavior of genes in adjacent stages considering a delay in order to make regulatory predictions. We applied the CDAA to iron deficiency microarray time course data from Dinneny et al. [2] to identify putative regulators involved in the control of known iron homeostasis transcription factors. Our results revealed distinct stages of the transcriptional response during 72 hours of exposure to iron deficient conditions. We identified transcription factors that are active within the first 12 hours of iron deficiency and experimentally validated their influence on 7 known iron transcription factors using quantitative real-time PCR (qRT-PCR). A majority (53%) of such influential predictions were validated, and one relationship was shown to be a direct binding interaction through yeast one-hybrid (Y1H) analysis. The CDAA was able to make testable and valid predictions that extend our understanding of the iron deficiency transcriptional cascade and can be used on comparable datasets to obtain a better understanding of regulatory responses in a variety of conditions.

## Results and Discussion

We developed the Cluster and Differential Alignment Algorithm (CDAA) to make testable predictions about regulatory influences based on time course transcriptome data. The CDAA contains three consecutive steps: Stage Separation, Gene to Stage Assignment, and Interaction Inference. These steps, implemented in MatLab source code for the CDAA (S1 File), delimit temporal stages of cascaded stress response, distribute differentially expressed genes across these stages based on expression activity, and identify potential regulations between genes in adjacent stages. The CDAA uses time course transcriptome data as an input and assumes that differential expression analysis has already been implemented based on specifics associated with the experimental approach (i.e. microarray [34] or RNA-Seq [35, 36]). It is important to note that as the CDAA operates solely on gene expression data, any posttranscriptional regulation will not be captured by its predictions. The algorithm starts by calculating normalized expression values to enforce compatibility across datasets obtained using different approaches:

$$\begin{array}{c} g_{i}\left(t_{k}\right)=\frac{g_{i}^{raw}\left(t_{k}\right)-{\bar{g}}_{i}^{raw}}{\sigma_{g_{i}^{raw}}},\;i=1,\ldots ,P,\;k=1,\ldots ,N, \end{array}$$ (1)

where $g_{i}^{raw}(t_{k})$ is the raw expression value of differentially expressed gene *i* at the *k*-th time point, ${\bar{g}}_{i}^{raw}$ and $\sigma_{g_{i}^{raw}}$ are the mean and standard deviation of the raw expression values, *P* is the number of differentially expressed genes, and *N* is the number of sampling time points.

### CDAA—Stage Separation

The first step of the CDAA separates a time course of all differentially expressed genes into distinct stages based on their transcriptional activity. This provides a mechanism to computationally assess the dynamic landscape of a transcriptional cascade. Stage separation is based on the assumption that transcriptional cascades are characterized by waves of activity, with early transcription factor activity (Initiation) triggering expression activity in subsequent stages (Response). Time intervals where groups of genes exhibit high expression activity can be identified and separated. The Stage Separation step of the CDAA assigns borders between dynamic stages by identifying the time interval where the majority of differentially expressed genes have their largest change in expression. This is based on the assumption that waves of expression activity increase in magnitude as they propagate until peak activity is reached.

The CDAA first normalizes changes in expression with respect to time using the difference in sample times to account for unevenly spaced time course data, typical in available time course datasets [2, 19, 20]. This allows the CDAA to compare small expression changes over small time intervals and large expression changes over large time intervals without bias. The normalized change in expression of gene *g*_*i*_ over the time interval (*t*_*k*_,*t*_*k*+1_) is defined as:

$$\begin{array}{c} s\left(g_{i},k\right)=\frac{g_{i}\left(t_{k+1}\right)-g_{i}\left(t_{k}\right)}{t_{k+1}-t_{k}},\;k=1,\ldots ,N-1. \end{array}$$ (2)

**Fig 1:**
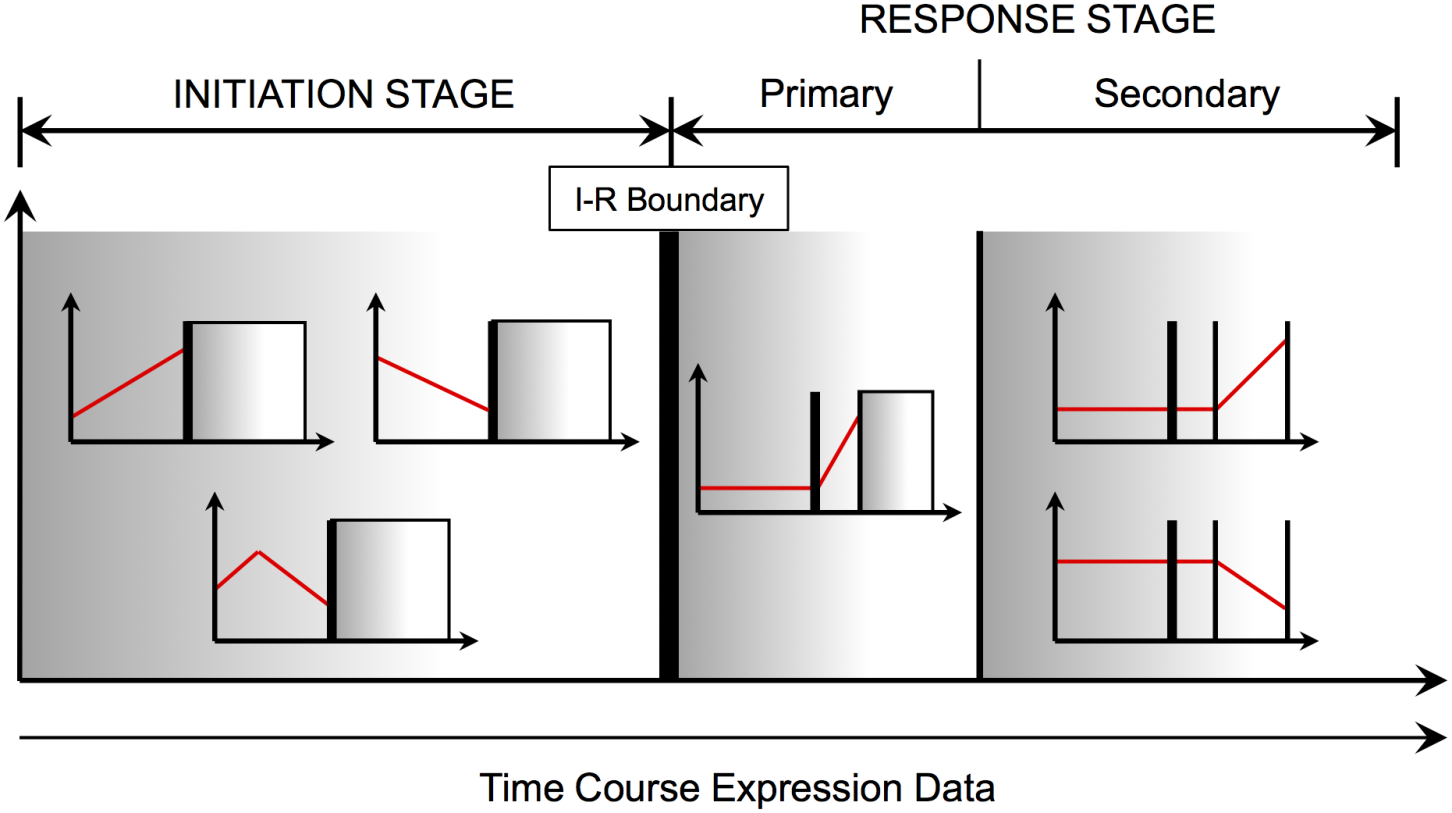
Gene to Stage Assignment. Genes active before the Initiation-Response (I-R) boundary are assigned to the INITIATION STAGE. Genes that start their activity after the I-R boundary are assigned to the RESPONSE STAGE. Primary response genes are active right after the I-R boundary and Secondary response genes are active later.

Each gene *g*_*i*_ is then assigned to a set 𝓖_*n*_, 1 ≤ *n* ≤ *N* − 1, if its maximum change in expression appears at the time interval (*t*_*n*_,*t*_*n*+1_) (*s*(*g*_*i*_,*n*) = max_*k* = 1,…,*N* − 1_ *s*(*g*_*i*_,*k*)). The set 𝓖_*b*_, 1 ≤ *b* ≤ *N* − 1, with maximum cardinality (number of elements) represents the time interval where the majority of genes have their highest activity, leading to assignment of the time boundary *t*_*b*_ at the time point preceding this interval. We refer to this boundary as the Initiation-Response (I-R) boundary. All time intervals to the left of the I-R boundary are denoted as the Initiation stage and all time intervals to the right of the I-R boundary are denoted as the Response stage. The Response stage can then be subdivided into Primary and Secondary response to account for genes that start exhibiting a change in expression directly after the I-R boundary or after some delay (Fig 1).

The approach above provides a systematic way of preliminarily partitioning genes based on the hypothesis that the activity of a few genes (Initiation stage) triggers later activity of a large set of genes (Response stage). The presence, characteristics, and duration of these stages will differ from process to process and dataset to dataset. The sampling scheme of the dataset will heavily influence the presence/duration of the Initiation stage, existence of a Secondary or even Tertiary response in the Response stage, and/or multiple I-R boundaries. The primary goal of this initial partitioning is to capture at least two distinct stages that would allow for later extraction of regulator (stage 1 gene) / target (stage 2 gene) interactions. A sampling scheme that results in less than two stages, which is highly unlikely to occur, would result in a dataset where regulator-target interactions would be difficult to predict.

### CDAA—Gene to Stage Assignment

The second step of the CDAA further characterizes activity in the Initiation, Primary Response, and Secondary Response stages and assigns genes to these stages based on their expression patterns. Genes are assigned to a specific stage based on the time intervals where expression activity for that gene is first seen. For example, genes active during the Initiation stage are classified as Initiation genes, regardless of their expression activity during subsequent stages. This assignment is determined using a stage specific clustering scheme. This scheme clusters expression values across the different stages, starting first with time points corresponding to the Initiation stage then iteratively adding time points from subsequent stages. This approach enables stage specific clustering, allowing for the effective partitioning of activity at each stage while eliminating the effect that dominant expression activity over small intervals can have on whole time course clustering. Clustering using Initiation stage time points starts by centering the expression values for all genes:

$$\begin{array}{c} g_{i}^{I}\left(t_{k}\right)=g_{i}\left(t_{k}\right)-{\bar{g}}_{i}^{I},\;i=1,\ldots ,P,\;k=1,\ldots ,b, \end{array}$$ (3)

where ${\bar{g}}_{i}^{I}$ is the mean gene expression value for gene *g*_*i*_ over time points *t*_*k*_ ≤ *t*_*b*_. The number of clusters chosen is not fixed and varies depending on the dataset. The ultimate goal of clustering is to partition genes into clusters that show activity during the Initiation stage and a cluster of genes that show little to no activity during the Initiation stage. This can be achieved heuristically or via cluster number defining techniques [37, 38]. Genes belonging to clusters with activity during the Initiation stage are assigned to the Initiation stage and genes with no apparent activity are assigned to the Response stage. Response stage genes are centered again, this time using expression values corresponding to Initiation and Primary Response stages:

$$\begin{array}{c} g_{j}^{R}\left(t_{k}\right)=g_{j}\left(t_{k}\right)-{\bar{g}}_{j}^{R},\;j=1,\ldots ,P-P_{I},\;k=1,\ldots ,b+c. \end{array}$$ (4)

Here *P*_*I*_ represents the number of genes assigned to the Initiation stage, ${\bar{g}}_{j}^{R}$ is the mean expression value for *g*_*j*_,*j* = 1,…,*P* − *P*_*I*_, over time points *t*_*k*_ ≤ *t*_*b*+*c*_, and *c* stands for the number of intervals in the Primary Response stage. Clustering is applied for a second time to isolate a group of genes with no activity after extending the time range. Genes belonging to active clusters are classified as Primary Response genes and genes belonging to the inactive cluster are Secondary Response genes. This incremental approach to clustering allows for the identification of waves of activity—the first wave containing clusters of genes active during the Initiation stage, the second wave containing clusters of genes whose activity starts at the Primary Response stage, and the final wave containing clusters of genes active only at the Secondary Response stage. This process can be adjusted based on the number of stages identified in the dataset.

### CDAA—Interaction Inference

The final step of the CDAA predicts putative regulatory relationships between genes in adjacent stages. This step is based on the assumption that the expression activity of regulator genes in one stage will be reflected in the expression activity of corresponding target genes in a subsequent stage with some delay in regulation [24, 26, 39]. Regulators are selected from genes classified in one particular stage and targets are selected from genes classified in the subsequent adjacent stage (i.e. Initiation and Primary Response). The Interaction Inference procedure uses changes in expression over time rather than expression values to assess trend similarities between putative regulators and targets. Changes in expression values are first normalized with respect to maximum change:

$$\begin{array}{c} s_{n}\left(g_{i},k\right)=\frac{s(g_{i},k)}{\max_{1\leq n\leq N-1}\left|s\left(g_{i},n\right)\right|},\;k=1,\ldots ,N-1. \end{array}$$ (5)

Here, *s*_*n*_(*g*_*i*_, *k*) is a signal that ranges from –1 to 1 over all *k*, where a value of –1 (or 1) corresponds to the largest negative (or positive) change.

The signal *s*_*n*_(*g*_*i*_, *k*) is discrete (one value represents an entire time interval), which limits the assessment of delayed similarities between a target gene, *g*_*T*_, and some putative regulator, *g*_*R*_. This problem is exacerbated when samples are sparse and non-uniform. The CDAA assigns values at intermediate time points by assuming that the change in expression is constant between sample time points. This assumption results in a zeroth-order approximation of *s*_*n*_(*g*_*i*_,*k*):

$$\begin{array}{c} s_{n}^{0}\left(g_{i},t\right)=s_{n}\left(g_{i},k\right),\;t_{k}<t\leq t_{k+1},\;k=1,\ldots ,N-1. \end{array}$$ (6)

Next, a dissimilarity score between the approximated expression change signal of a candidate regulator, $s_{n}^{0}(g_{R},t)$, and a delayed (shifted) version of the approximated expression change signal of a candidate target, $s_{n}^{0}(g_{T},t+\Delta t)$, is calculated using a modification of pattern alignment technique [7, 40]. A smaller dissimilarity score corresponds to a higher chance that the behavior in the regulator influences the expression activity of the target. Dissimilarity scores are calculated for a candidate pair, (*g*_*R*_, *g*_*T*_), for a set of delays:

$$\begin{array}{c} d\left(g_{R},g_{T},m\Delta T\right)=\frac{1}{M}\sum_{t_{i}\in T}\left|s_{n}^{0}\left(g_{R},t_{i}\right)-s_{n}^{0}\left(g_{T},t_{i}+m\Delta T\right)\right|,\;m=0,1,\ldots ,M-1, \end{array}$$ (7)

where 𝓣 is the set of time points in the regulator’s stage, Δ*T* is the largest common divisor of the time intervals in the time course data, and M represents the maximum number of Δ*T* that can fit in each time interval corresponding to regulator’s and target’s stages. The resulting dissimilarity score quantifies likelihood of a positive influence between a regulator and its target assuming similar, yet delayed, expression behavior. Dissimilarity scores for the inverted regulator expression $\hat{d}(g_{R},g_{T},m\Delta T)$ are also calculated to detect possible negative influences. The smaller of *d*(*g*_*R*_,*g*_*T*_,*m*Δ*T*) and $\hat{d}(g_{R},g_{T},m\Delta T)$ is taken for each time delay *m*Δ*T*, and the predicted influence type (positive or negative regulation) is recorded. Dissimilarity scores for a potential target are organized into a dissimilarity table where rows correspond to potential regulators and columns to delays. Rows at which the minimal dissimilarity score is achieved at a delay of 0 hrs are discarded to avoid assigning a regulatory connection between genes that are co-expressed.

Noise in expression data can often disrupt the accuracy of alignment algorithms [18]. The algorithm addresses the possibility that some small changes in gene activity may be due to experimental error or noise by applying thresholding to normalized gene expression changes, $s_{n}^{0}(g_{i},m\Delta T)$, to convert changes into events of upregulation (1), downregulation (–1), or no regulation (0) [24]:

$$\begin{array}{c} s_{n,thr}^{0}\left(g_{i},t_{j}\right)=\{\begin{array}{c} 1,\;\text{if}\;s_{n}^{0}\left(g_{i},m\Delta T\right)>thr,\\ 0,\;\text{if}\;|s_{n}^{0}\left(g_{i},m\Delta T\right)|<thr,\\ -1,\;\text{if}\;s_{n}^{0}\left(g_{i},m\Delta T\right)<-thr. \end{array} \end{array}$$ (8)

Dissimilarity tables for multiple thresholded versions of the signal $s_{n,thr}^{0}(g_{i},m\Delta T)$ along with the unthresholded version, $s_{n}^{0}(g_{i},m\Delta T)$, are generated. Different threshold values assume different levels of noise and will result in different dissimilarity tables for the same potential target. A maximum dissimilarity cutoff is used to identify candidate regulators that are more likely to influence a potential target at each threshold. Consensus over multiple thresholds results in CDAA regulatory predictions that can be experimentally validated.

### Application of the CDAA

**Fig 2:**
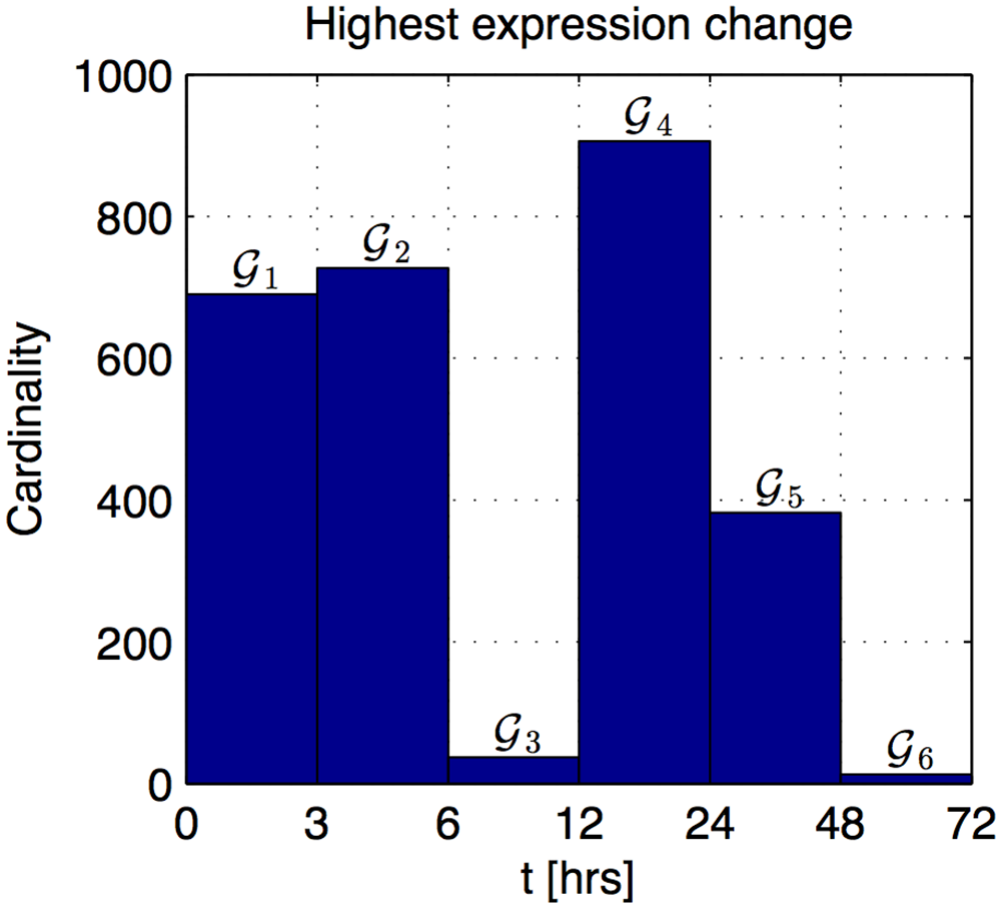
Number of genes in each gene set (cardinality). Gene set 𝓖_*n*_, *n* = 1,…,6, is comprised of genes whose maximum change occurs over the interval (*t*_*n*_, *t*_*n*+1_).

We applied the CDAA to the iron deficiency dataset from Dinneny et al. [2] with *P* = 2754 differentially expressed genes sampled at *N* = 7 time points in *Arabidopsis thaliana* roots 0, 3, 6, 12, 24, 48, and 72 hours after exposure to iron deficient conditions (S2 File). Differentially expressed genes were defined in Long et al. [4] as genes that were at least 1.5-fold differentially regulated with a false discovery rate (Q-value) less than 10^−4^. We maintained this designation for application of the CDAA. We calculated changes in expression for each differentially expressed gene using Eq (2) and assembled the sets 𝓖_*n*_,*n* = 1,…,6, with genes whose maximum change occurs over the interval (*t*_*n*_, *t*_*n*+1_). The number of genes in each set 𝓖_*n*_ (cardinality) is shown in Fig 2. The set 𝓖_4_, corresponding to the interval between 12 and 24 hrs, contains the maximum number of genes. We assigned the I-R boundary to the time point preceding this interval, *t*_*b*_ = 12 hrs (*b* = 4), and defined the stages as Initiation: 0 ≤ *t* ≤ 12 hrs and Response: 12 < *t* ≤ 72 hrs. We assigned Primary Response (defined as the interval of high activity following the I-R boundary) to 12 < *t* ≤ 24 hrs. The transcriptional iron deficiency response as described by the CDAA, therefore, has at least 3 waves of activity, with the first wave ending at 12 hours.

**Fig 3:**
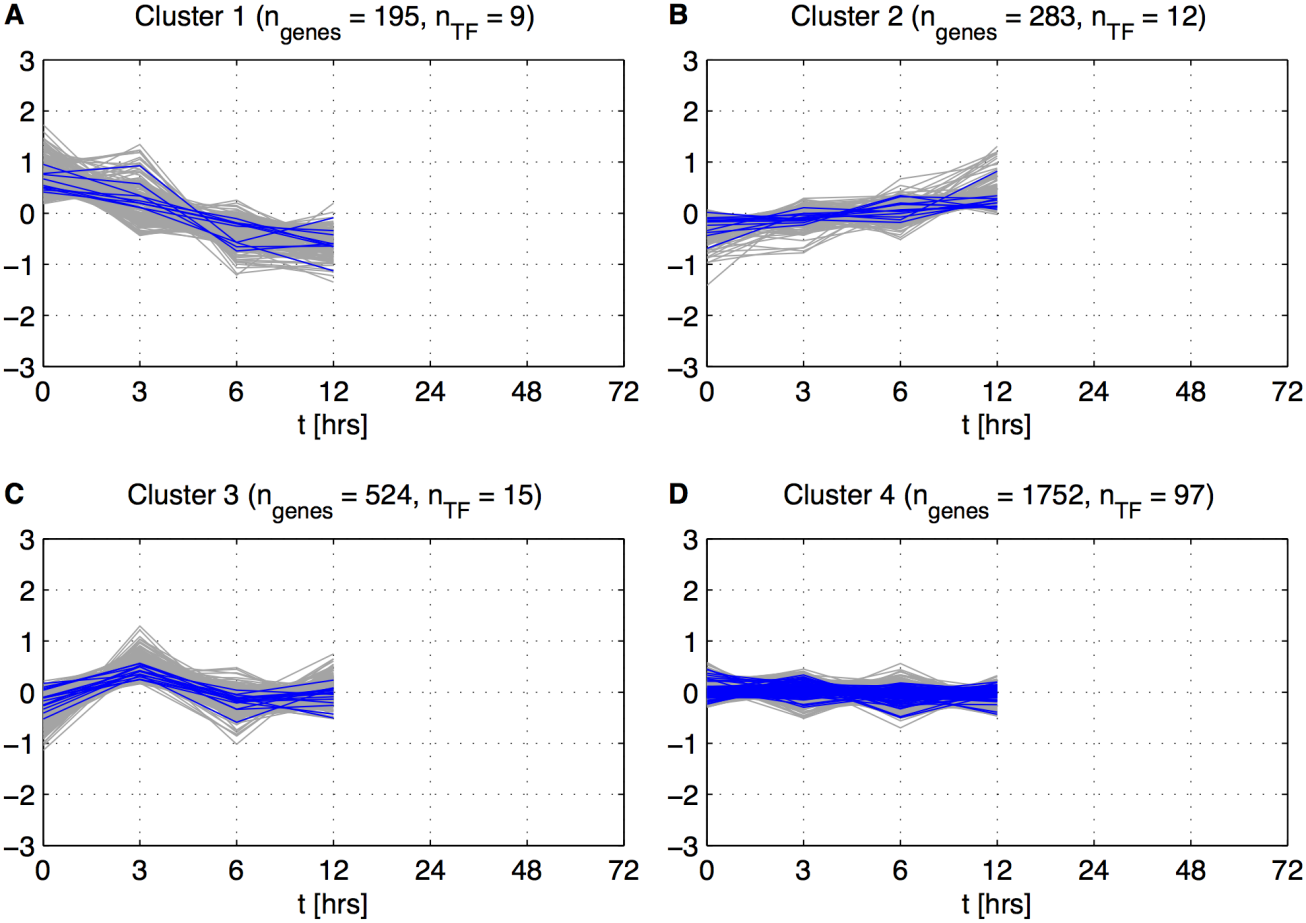
4 points based clustering. Clustering based on centered expression values $g_{i}^{I}(t_{k}),\;i=1,\ldots ,2754,\;k=1,\ldots ,4$. *n*_*genes*_—number of genes in each cluster and *n*_*TF*_—number of Transcription Factors in each cluster.

After the Stage Separation step, we clustered all differentially expressed genes based on expression patterns during the Initiation stage for Gene to Stage Assignment (Fig 3). We chose k-means clustering for this procedure since it is not as computationally intensive as hierarchical clustering or self-organizing maps but is shown to produce similar results when applied to transcriptome datasets [38]. Clustering revealed four behavioral patterns: decrease in expression (Fig 3A), increase in expression (Fig 3B), oscillatory behavior (Fig 3C), and no change in expression (Fig 3D). We assigned genes from Clusters 1 through 3 to the Initiation stage, and used the inactive Cluster 4 for the second round of clustering. Cluster 4 contained 1752 genes (63% of all genes in the dataset) and 97 known transcription factors (72% of all known transcription factors in the dataset). Hence, the majority of activity associated with iron deficiency occurs after 12 hours of exposure. Clusters 1 through 3 contain 36 transcription factors, none of which have so far been implicated in the iron deficiency response, meaning that these regulators may trigger the plant’s overall response to the stress. The list of genes annotated as transcription factors is shown in S3 File.

**Fig 4:**
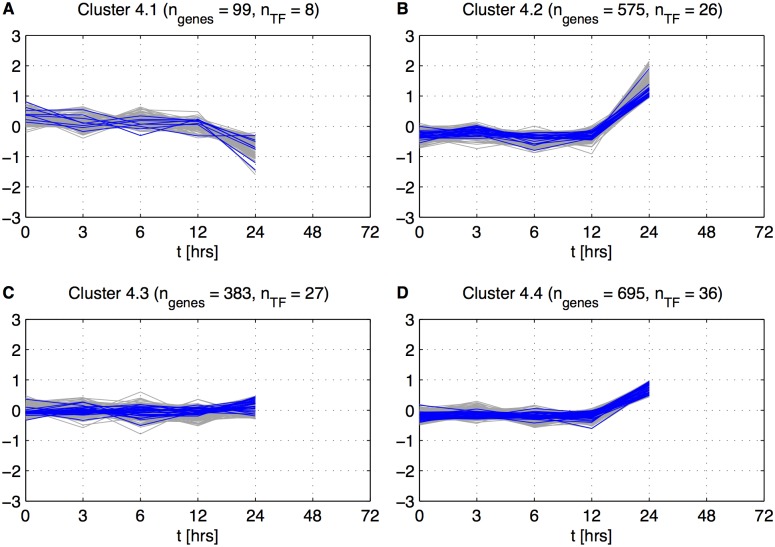
5 points based subclustering. Clustering based on centered expression values $g_{j}^{R}(t_{k}),\;j=1,\ldots ,1752,\;k=1,\ldots ,5$. *n*_*genes*_—number of genes in each cluster and *n*_*TF*_—number of Transcription Factors in each cluster.

We added the Primary Response stage time point *t*_5_ = 24 hrs to the expression patterns to classify the remaining genes. The results of clustering applied to Cluster 4 after adding the 24 hrs time point are shown in Fig 4. Genes from Clusters 4.2 and 4.4 show a rise in expression after 12 hours, genes from Cluster 4.1 show a decrease in expression, and genes from Cluster 4.3 are inactive during the whole interval from 0 to 24 hours. Thus, we assigned genes from Clusters 4.1, 4.2, and 4.4 to the Primary Response stage. Cluster assignments for each gene are listed in S4 File.

We selected 7 transcription factors with published roles in the iron deficiency response and used the Interaction Inference stage of the CDAA to predict relationships involving these genes as a means of focusing validation to a feasible set. We were limited to genes that were present in the Affymetrix chip used for this particular expression analysis. Therefore, transcriptome data for the master iron deficiency regulator *FIT1* and its heterodimer partner *bHLH38* were not available for analysis by the CDAA. The known iron homeostasis transcription factors chosen for this study were *bHLH39* [14], *bHLH101* [15], *bHLH115* [4], *PYE* [4], *MYB10* [6], *MYB72* [6], and *BTS* [4, 16].

All 7 known iron related transcription factors were assigned to the Primary Response stage by the CDAA (*bHLH39*, *bHLH101*, and *bHLH115* appeared in Cluster 4.2 and the remaining transcription factors appeared in Cluster 4.4). We hypothesized that regulators from the Initiation stage (Clusters 1–3) may be responsible for influencing the known iron homeostasis transcription factors. Since the Initiation stage regulators and known iron transcription factors appeared in adjacent stages, we applied the CDAA to test this hypothesis.

We calculated normalized changes in expression for each transcription factor from the Initiation stage (regulator) and each known iron transcription factor (target). The largest common divisor for time intervals in Initiation and Response stages is 3 hours, so this value served as the delay step size (i.e. Δ*T* = 3 hours). Since each stage is 12 hours long, a maximum of 4Δ*T* can fit in each stage (i.e. *M* = 4).

**Fig 5:**
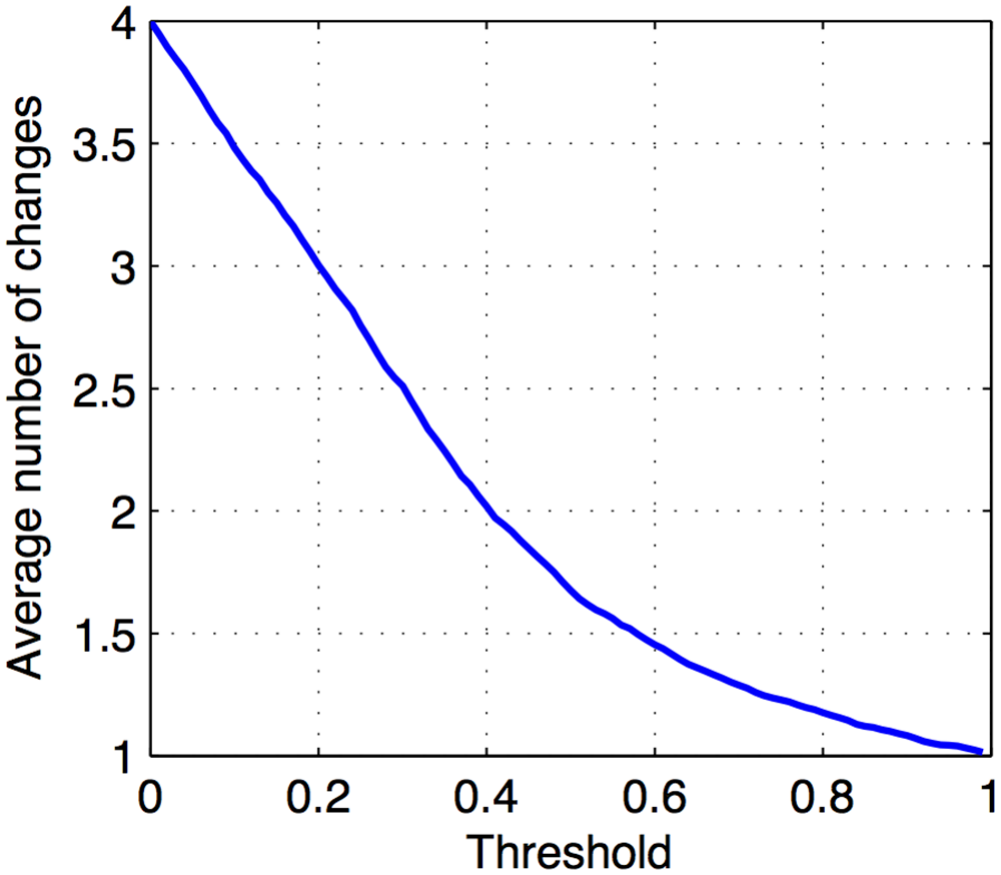
Average number of changes above the threshold per gene. Changes in expression (*s*_*n*_(*g*_*i*_, *k*), *k* = 1,…,4) for Initiation stage genes were thresholded with a range of cutoff values. The graph shows the average number of changes that exceed the threshold per gene out of 4 possible changes.

We selected thresholds for the noise reduction portion of the Interaction Inference step to account for different levels of signal fluctuations. We first applied a set of thresholds to Initiation Stage gene expression changes, *s*_*n*_(*g*_*i*_, *k*), *k* = 1,…,4, to obtain the average number of changes per gene above the threshold (Fig 5). Based on these results, we set thresholds equal to 0.2 and 0.4 so that 25% and 50% of possible changes per gene, respectively, were attributed to noise. Using these thresholds, we produced two more versions ($s_{n,0.2}^{0}(g_{i},m\Delta T)$ and $s_{n,0.4}^{0}(g_{i},m\Delta T)$) of the normalized change in expression signal $s_{n}^{0}(g_{i},m\Delta T)$ for each regulator from the Initiation stage and each known iron transcription factor.

We calculated dissimilarity values between all regulators from the Initiation stage and one of the targets, *PYE*, at multiple time delays for each threshold and organized them into dissimilarity tables (S1 Fig). We chose a cutoff of 0.4 to remove potential regulators with high dissimilarity over all delays. This cutoff produces a testable number of predicted regulators (3–5) per target. A deviation by 0.1 from this value adds or eliminates 1 to 3 candidate regulators. The 4 regulators that appeared in 2 out of 3 dissimilarity tables were assigned as potential regulators of *PYE* (Table 2).

**Table 2:** *PYE* dissimilarity tables summary.

**Regulator**	**Differential pattern**		
	No Thr.	Thr. = 0.2	Thr. = 0.4
ASIL2	✔	✔	✔
ETF9	✔		✔
WRKY57	✔		✔
MYB55	✔	✔	
GNU1		✔	
TG		✔	
LRL3		✔	
WRKY26		✔	
RD26		✔	
COL4		✔	✔
TGA2			✔
OBP4			✔

Table lists regulators that appeared in dissimilarity tables for each thresholded version of expression patterns. Regulators that appeared for at least 2 patterns (ASIL2, ETF9, WRKY57, MYB55, and COL4) were identified as potential regulators of *PYE*.

**Fig 6:**
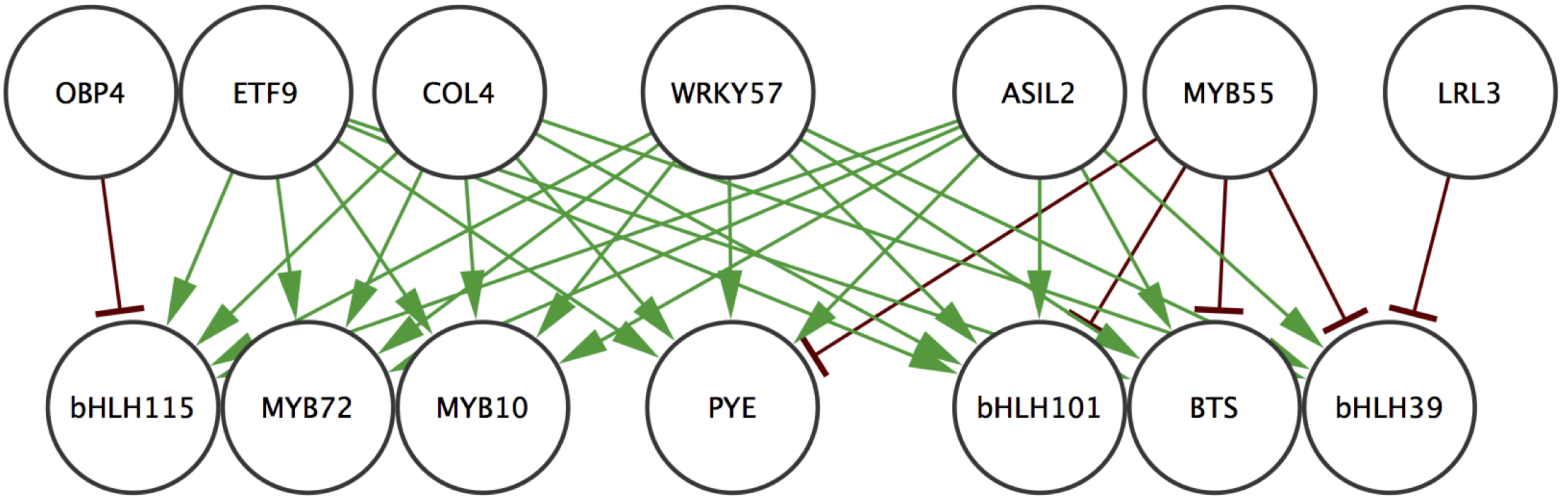
Regulatory relationships predicted by the CDAA. Predicted regulations between 7 early stage transcription factors and 7 known iron homeostasis transcription factors. Edges indicating positive regulations are green and edges indicating negative regulations are red.

Using the same procedure, we determined potential regulators for the remaining targets, for which dissimilarity tables are shown in S5 File. All 7 targets were predicted to be regulated by a set of 7 regulators. These predictions resulted in a small network of interactions containing 14 nodes and 32 edges (Fig 6). The majority of the edges (26 out of 32) were predicted to be positive regulations. 6 of the 7 regulators are named genes, though only 3 have been characterized (WRKY57 [41, 42], ASIL2 [43, 44], and LRL3 [45]) *and none are currently linked to iron homeostasis*. The remaining regulator (At2g36720) was named Early Transcription Factor 9 (ETF9).

### Validation of predicted relationships

**Fig 7:**
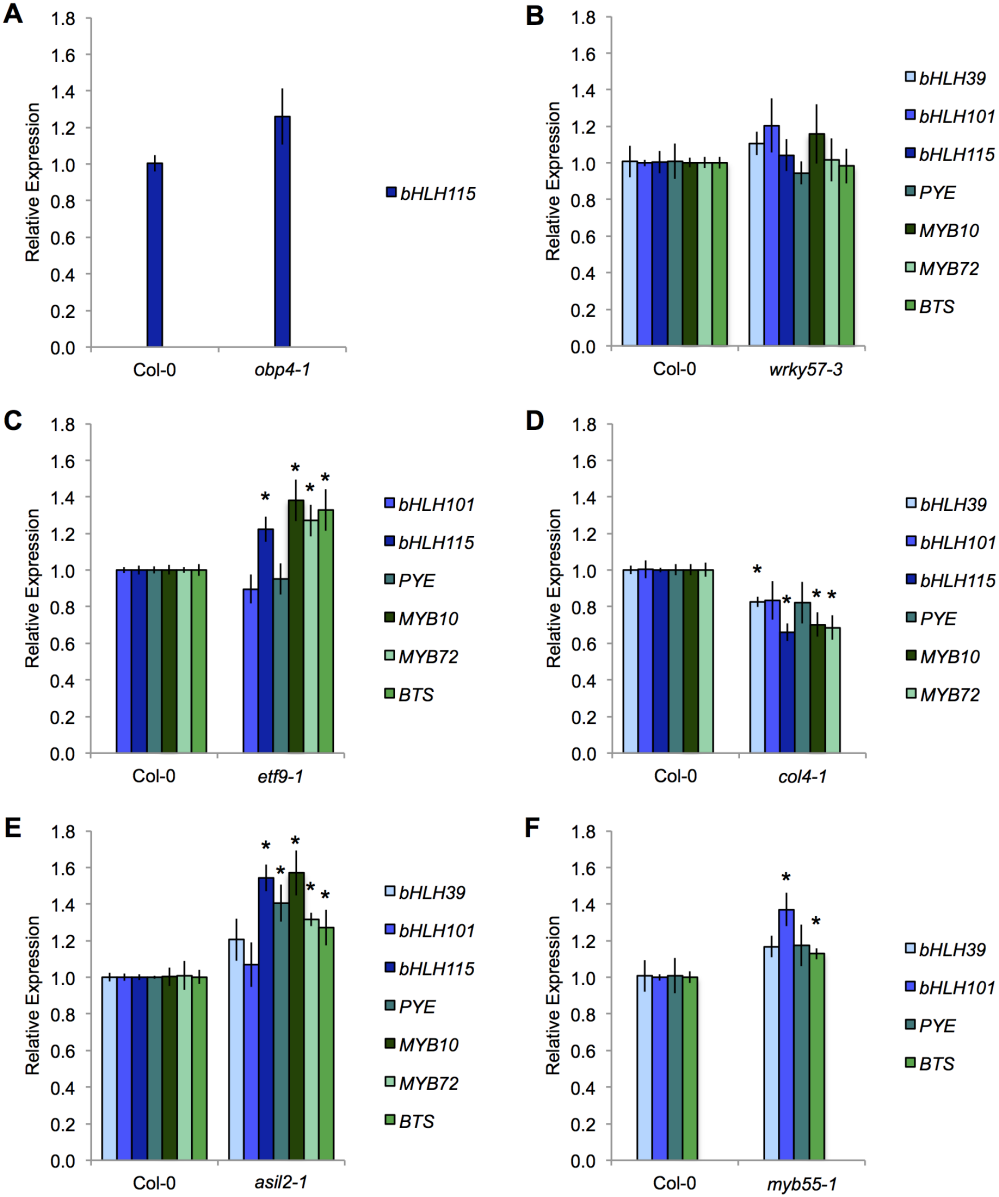
Expression validation of predicted targets in mutant regulator backgrounds. Root tissue was collected from seedlings grown 4 days on iron sufficient media and transferred to iron deficient media for 3 days. Expression values are normalized to **β*-tubulin* and to WT (Col-0) expression for each gene. Error bars indicate ±SEM (n = 4). Mutant backgrounds are (A)*obp4-1*, (B)*wrky57-3*, (C)*etf9-1*, (D)*col4-1*, (E)*asil2-1*, and (F)*myb55-1*. Asterisk indicates significant difference from WT (Student’s t-test, *p* < 0.05).

We used quantitative real time PCR (qRT-PCR) to validate predicted regulator-target relationships by measuring transcript of targets in a background with significantly altered expression of the predicted regulator. Ideally, multiple mutant alleles could be tested for each regulator, but due to limited availability of lines with significantly altered expression, only one mutant allele per regulator was tested (with the exception of ASIL2 for which 2 lines were tested and LRL3 for which no suitable line was identified during validation). We sequenced insertion locations; 4 are exonic (*etf9-1*, *asil2-1*, *myb55-1*, and *asil2-2*), 2 are intronic (*wrky57-3* and *col4-1*), and 1 is in a promoter (*obp4-1*) (S2 Fig). Insertions in the introns and promoter led to reduced regulator expression and no full product was made in mutants with exon insertions (S3 Fig, S4 Fig, S5 Fig). We measured transcript levels of predicted targets for each regulator in the mutant backgrounds as compared to wild-type (either Col-0 or L*er*) in 7 day old seedlings, 3 days after shift from iron sufficient to deficient media (Fig 7, S4 Fig). We considered target expression significantly affected if it differed from wild-type values with a p-value of 0.05 or less. We considered significantly altered target expression in either direction as support for an influential relationship and considered altered expression in the correct direction (i.e. lower target expression in the mutant of a predicted positive influencer) as support for a specific type of influential relationship. In the case of ASIL2, for which 2 mutant lines were available, we considered significantly affected expression in either mutant line as support.

Based on qRT-PCR results, we were able to validate 17 out of 32 influential relationships (53%) (S6 Fig). Interestingly, though a majority of influential relationships were validated, some of the type specific (activation or inhibition) predictions were incorrect. This may be because the CDAA was based on the assumption that change in expression of a potential regulator leads to change in the expression of its target gene in isolation (a rise in a target can only be the result of a rise in its regulator if these genes have a positive influential relationship). This assumption does not take into account combinatorial effects of multiple transcription factors acting on the expression of one gene, and the resulting algorithm predictions of positive or negative influence are unable to assess to what extent each regulator controls the expression of each target in combination. Also, it may be possible that similar yet delayed expression patterns could instead be indicative of a regulator acting to continuously dampen expression of targets that are activated by another regulator. This effect is a likely explanation for the results seen for ETF9 and ASIL2, which both were predicted as positive regulators of their targets by the CDAA due to trend similarities. Experimentally measured expression of regulators and targets using qRT-PCR indicate, however, that target expression is increased in both mutant backgrounds. This may indicate that under iron deprivation, increased expression of the regulators works to constrain the expression of the targets. Thus, the assumption on the type of influence between a potential regulator and its target, widely used in gene regulatory network inference algorithms, appears to be limited in the case of our application.

Transcription factors that were not predicted to be regulated by the 7 regulators (dissimilarity value higher than 0.4 at at least 2 out of 3 thresholds) were chosen as negative control genes. Expression of each negative control gene was not significantly different in the mutant backgrounds, indicating that the expression alterations seen are specific to the predictions of the algorithm and not indicative of widespread expression alterations in the mutants (S4 Fig, S7 Fig).

While other algorithms have been developed to infer regulatory relationships based upon transcriptomic data, they are typically driven by substantial prior knowledge of regulator-target relationships or are of limited utility for minimal, unevenly spaced datasets. For example, the Event Method [24], similar to the CDAA, aims to infer causal relationships between genes by aligning their differential expression patterns with an assumption of a possible delay in regulation, but required modification to work with an unevenly sampled time course dataset. After implementing a linear interpolation step as a modification to the Event Method algorithm and limiting a set of genes to transcription factors, we obtained predictions for the same known iron response genes used with the CDAA. The predictions resulted in a network containing 44 nodes and 144 edges. Only 2 regulatory connections that were identified by the CDAA and experimentally validated were found in the Event Method prediction set. Thus, the CDAA is an improvement on currently available regulatory inference algorithms.

### Identification of direct connections using enhanced yeast one-hybrid

Though the CDAA can predict influential relationships between transcription factors and their targets, it can not differentiate between direct (binding) or indirect connections. We utilized yeast one-hybrid (Y1H) analysis to identify direct regulatory connections involving one of the target genes, *PYE*, and to see if any of these connections correspond to CDAA predictions. We cloned the promoter region of *PYE* into Y1H reporter constructs and screened it against an expanded collection of *A. thaliana* root specific transcription factors [46, 47]. We identified 20 transcription factors that bind to the *PYE* promoter (S1 Table). Two of these transcription factors are differentially expressed under iron deficiency and were thus a part of CDAA analysis. It is likely that other interactions could have been missed in the Y1H analysis because this assay is conducted *in vitro* and independent of iron availability. Some direct interactions may require other regulatory machinery found only in plants or only under iron deficiency.

One of the two iron-responsive transcription factors that bound the *PYE* promoter is ASIL2, which was predicted and validated to affect the expression pattern of *PYE* (Fig 7, S4 Fig). Interestingly, ASIL1, the close homolog to ASIL2, also binds the *PYE* promoter (S1 Table). The other iron-responsive transcription factor that targets *PYE*, HB-12, was not predicted to regulate *PYE* expression via the CDAA because the minimum dissimilarity in the alignment of *HB-12* and *PYE* expression occurred at a delay of 0 hrs, where the CDAA is unable to distinguish between genes affecting each other and genes that are co-expressed.

The close homolog to ASIL2, ASIL1, is known to bind to the GT-box-like-element (GTGATT) [48]. This element is found in the *PYE* promoter region. Given that *PYE* was validated as a direct connection, it is possible that ASIL1 and ASIL2 share this binding element. It could also be possible that ASIL2 binds to other unidentified promoter elements. The CDAA as an expression analysis tool will therefore be particularly effective in tandem with promoter analysis and high throughput transcription factor binding data including Y1H and chromatin immunoprecipitation sequencing (ChIP-Seq). These additional experiments could improve the specificity of further predictions by revealing characteristics that are common specifically to direct connections. It is striking that even though binding predictions were not the immediate goal of the CDAA, one such connection was detected.

The 7 regulators predicted to influence known iron regulators come from distinct transcription factor families and are all previously unlinked to iron homeostasis. Several of the validated transcription factors (S6 Fig) have known or predicted roles in stress and development. COL4 (At5g24930) has a predicted B-box zinc finger domain and CCT motif [49]. Although COL4 is uncharacterized, it is closely related to COL3, involved in light signaling and root growth [50]. ASIL2 (At3g14180) has been shown to play a role in regulating embryo maturation together with its close homolog ASIL1 [43]. Both ASIL1 and ASIL2 are members of the trihelix transcription factor family. ASIL1 recognizes and binds to a specific element in promoter sequences, and over 1000 genes are misregulated in the *asil1-1* mutant background [48]. Early chlorophyll accumulation during embryo development is seen in both *asil1* and *asil2* mutants, and more strongly in an *asil1asil2* double mutant [43]. Given the requirements of iron for chlorophyll biosynthesis, as well as links between seed iron content and embryogenesis [16, 51–53], it is possible that ASIL2’s role in embryo development is related to its role in iron homeostasis.

We did not observe visual phenotypic differences from wild type for any of the mutants when grown under iron deficient conditions (data not shown). This result is not necessarily unexpected, especially given the modest alterations in target expression seen. The algorithm assigns multiple regulators to each iron homeostasis gene of interest, indicating that combinatorial effects may be in effect. Therefore, it will likely be necessary to examine higher order mutants to observe more dramatic phenotypes.

### Conclusion

**Fig 8:**
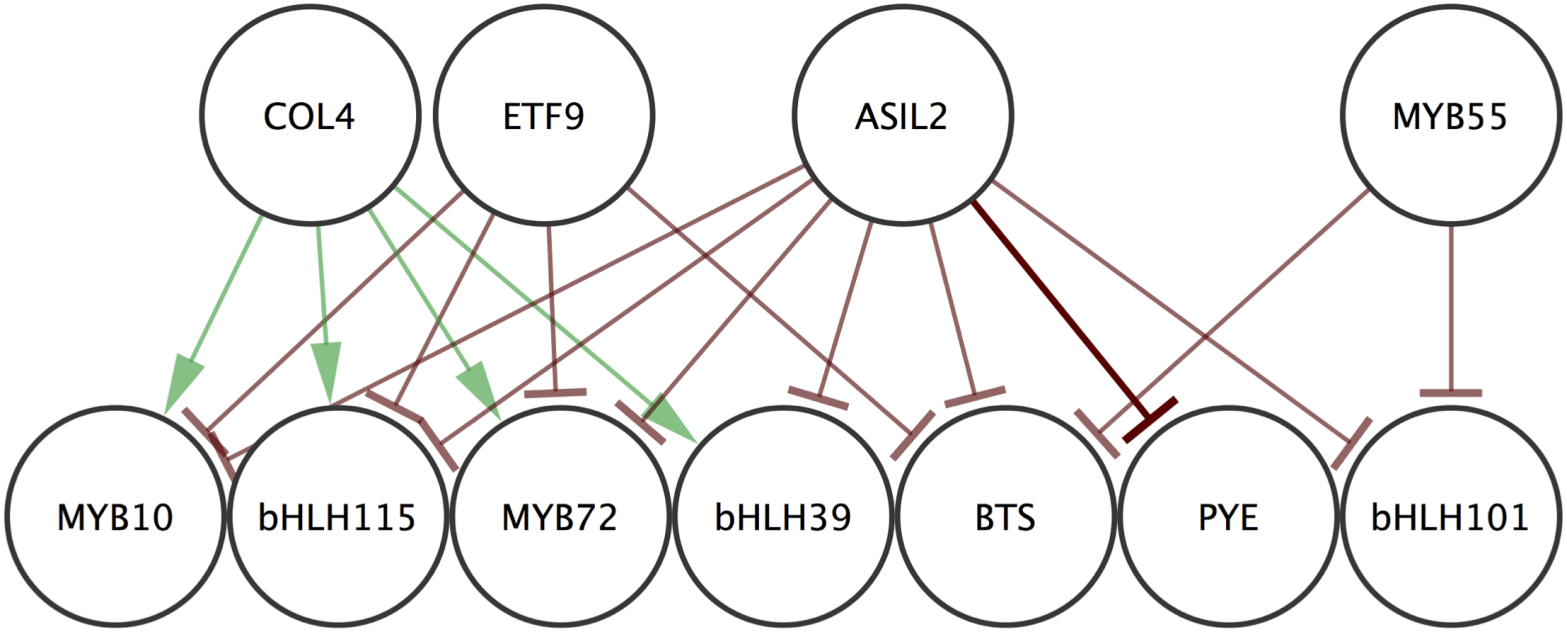
Experimentally validated regulatory relationships. Validated regulations between 4 early stage transcription factors and 7 known iron homeostasis transcription factors. Edges indicating positive regulations are green and edges indicating negative regulations are red. Edge indicating a direct connection validated by Y1H is darker.

The CDAA was able to make specific predictions about regulatory relationships between genes. A set of 931 potential regulatory relationships between 133 differentially expressed transcription factors and the 7 chosen targets was reduced by the CDAA to a very testable subset of 32 connections. The majority of the relationship predictions (53%) were experimentally validated by significantly altered target expression in a background with altered regulator expression. *The regulators identified were previously unlinked both to a role in iron deficiency and to the predicted targets*. One of the connections predicted by this algorithm was a direct connection, validated by Y1H analysis. Together, these results yield a small network of interactions which has expanded our understanding of the iron deficiency response in *A. thaliana* to novel genes and connections (Fig 8). Thus, the developed CDAA is capable of making predictions with biological significance and can be used to reveal gene regulatory connections in distinct fields of study.

## Materials and Methods

### Plant Growth and Materials

The *Arabidopsis thaliana* ecotypes Columbia (Col-0) and Landsberg *erecta* (L*er*) were used as wild type, depending on mutant background. T-DNA insertion lines for *obp4-1* (SALK_118463), *wrky57-3* (GK-078H12), *etf9-1* (SALK_025328), *col4-1* (SALK_092012C), *asil2-1* (SAIL_258_F06), *myb55-1* (GK-460G09), and *asil2-2* (ET8777) were confirmed using primers listed in S2 Table. *A. thaliana* seedlings were grown on iron sufficient media (+Fe) containing Murashige and Skoog basal salt solution supplemented with 0.05% (w/v) MES, 1% (w/v) sucrose, and 0.1 mM Fe-EDTA in the place of iron sulfate. Iron deficient media (–Fe) is prepared as described above except 0.3 mM of the iron chelator ferrozine is substituted for iron sulfate. Prior to plating, seeds were sterilized in 70% EtOH for 5 minutes, 30% bleach and 0.02% Triton X-100 for 15 minutes, and then rinsed 3 times in dH_2_O. Seeds were stratified in dH_2_O for 2–3 days at 4°C. For expression analysis, seeds were sown directly on 100 *μ*m Nitex Nylon mesh (Genesee Scientific) on square plates filled with iron sufficient media for 4 days, and transferred to iron deficient media for 3 days.

### qRT-PCR

Total RNA was isolated from pooled roots of *A. thaliana* seedlings using the RNeasy Plant Mini Kit (Qiagen). cDNA was synthesized using the SuperScript® III cDNA synthesis kit (Life Technologies) with oligo(dT) primers. qRT-PCR was performed using iTaq™ Universal SYBR® Green Supermix (Bio-Rad) and the StepOnePlus™ Real-Time PCR System (Applied Biosystems). Primers are listed in S2 Table. Relative expression was calculated using the $2^{-\Delta \Delta C_{T}}$ method, normalized to **β*-tubulin* and wild type. Statistical analysis was performed using Student’s t-test (*p* < 0.05) (n = 4).

### Plasmid Construction

The *PYE* promoter construct was created as described in Long et al.[4]. Briefly, 1120 bp upstream of the *PYE* start codon was amplified using primers listed in S2 Table and cloned into the pDONR™ P4-P1R (Invitrogen) vector. This fragment was recombined into HIS3 and LacZ promoter:reporter vectors for enhanced yeast one-hybrid (Y1H) screening, as described in Gaudinier et al. [47].

## Supporting Information

S1 FigDissimilarity tables for PYE at different thresholds.Dissimilarity scores between PYE and its putative regulators at a range of delays based on differential expression patterns with (A) No threshold, (B) Threshold of 0.2, or (C) Threshold of 0.4. ‘-i’ signifies that smaller dissimilarity scores were obtained for inverted regulator expression ($\hat{d}(g_{R},g_{T},m\Delta T)<d(g_{R},g_{T},m\Delta T)\;\forall m$); ‘-a’ signifies that smaller dissimilarity scores were obtained for non-inverted regulator expression ($d(g_{R},g_{T},m\Delta T)<\hat{d}(g_{R},g_{T},m\Delta T)\;\forall m$).(TIFF)

S2 Fig Location of T-DNA insertions and qRT-PCR primers in regulator genes. Regulator genes shown with exons in blue, untranslated regions (UTR) in gray, and promoters and introns as lines. Insertion locations are indicated with triangles and lines underneath genes indicate region spanned by qRT-PCR primers.
(TIFF)

S3 Fig Expression of regulators in mutant backgrounds. Root tissue was collected from seedlings grown 4 days on iron sufficient media and transferred to iron deficient media for 3 days. Expression values are normalized to **β*-tubulin* and to WT (Col-0) expression for each gene. Error bars indicate ±SEM (n = 4). Expression of (A) *OBP4*, (B) *WRKY57*, (C) *ETF9*, (D) *COL4*, (E) *ASIL2*, and (F) *MYB55* in respective mutant regulator backgrounds. Asterisk indicates significant difference from WT (Student’s t-test, *p* < 0.05).
(TIFF)

S4 Fig Expression validation in alternate allele *asil2-2*. Root tissue was collected from seedlings grown 4 days on iron sufficient media and transferred to iron deficient media for 3 days. Expression values are normalized to **β*-tubulin* and to WT (L*er*) expression for each gene. Error bars indicate ±SEM (n = 4). Expression of (A) *ASIL2* regulator, (B) ASIL2 targets, and (C) negative control gene *IAA27* in *asil2-2* mutant background. Asterisk indicates significant difference from WT (Student’s t-test, *p* < 0.05).
(TIFF)

S5 Fig No accumulation of full-length transcript in exonic insertions. Root tissue was collected from seedlings grown 4 days on iron sufficient media and transferred to iron deficient media for 3 days. PCR was performed on cDNA using primers for full length product (TOPO F&R) for (A) *ETF9*, (B) *ASIL2*, and (C) *MYB55*, each shown with **β*-tubulin* (*bTUB*) transcript as a control and run until saturation (35 cycles).
(TIFF)

S6 Fig Predicted and tested relationships between regulators and known iron homeostasis transcription factors. (TIFF)

S7 Fig Expression of negative control genes in mutant backgrounds. Root tissue was collected from seedlings grown 4 days on iron sufficient media and transferred to iron deficient media for 3 days. Expression values are normalized to **β*-tubulin* and to WT (Col-0) expression for each gene. Error bars indicate ±SEM (n = 4). Expression of (A) *ERF3*, (B) *IAA27*, (C) *IAA27*, (D) *UPB1*, (E) *IAA27*, and (F) *UPB1* negative control genes in mutant regulator backgrounds. All values are not significantly different from WT (Student’s t-test, *p* < 0.05).
(TIFF)

S1 Table Transcription factors that bind to the *PYE* promoter in Y1H analysis. Transcription factors with gene activity under iron deficiency are indicated in red and the connection predicted by the CDAA is indicated in bold.
(TIFF)

S2 Table Primers used in this study. (TIFF)

S1 File MatLab source code for the CDAA. (M)

S2 File Transcriptome data. (CSV)

S3 File List of transcription factors. (CSV)

S4 File Gene cluster membership. (CSV)

S5 File Dissimilarity tables for targets. (PDF)
