# Supplementary figures and images for: Clustering and Differential Alignment Algorithm: Identification of Early Stage Regulators in the Arabidopsis thaliana Iron Deficiency Response
Source: PLoS One. 2015 Aug 28;10(8):e0136591. doi: 10.1371/journal.pone.0136591 (PMC4552565; doi:10.1371/journal.pone.0136591)

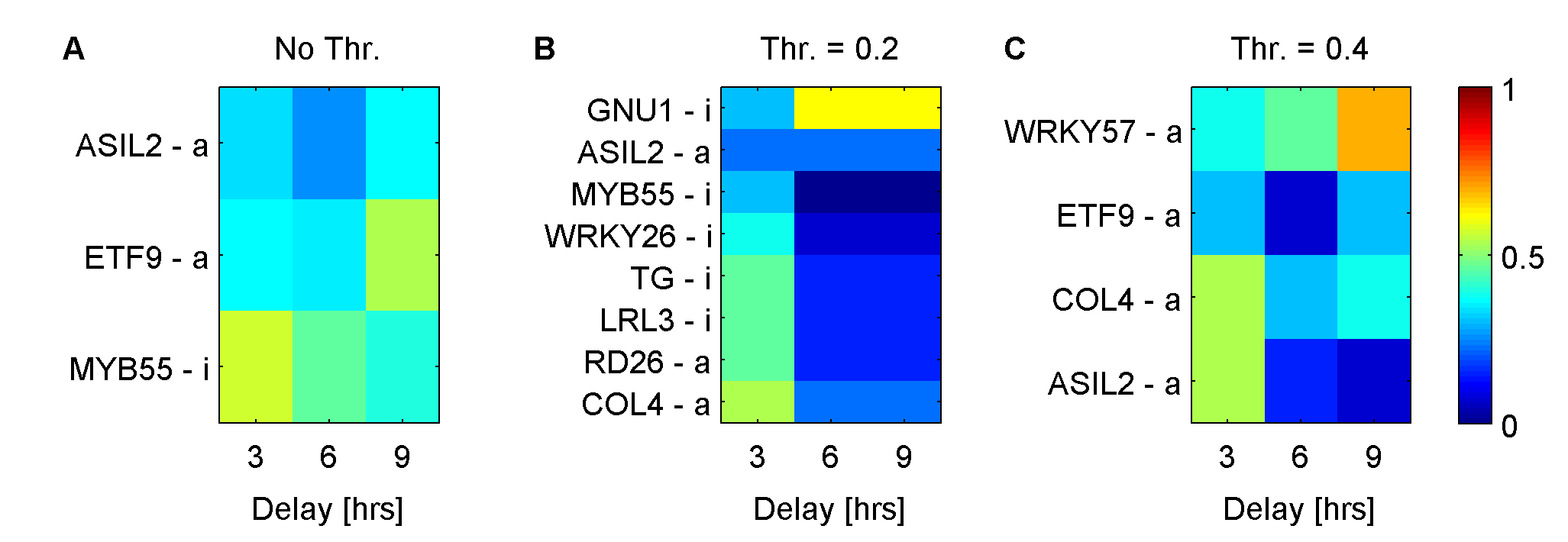

Supplement: S1 Fig — Dissimilarity scores between PYE and its putative regulators at a range of delays based on differential expression patterns with (A) No threshold, (B) Threshold of 0.2, or (C) Threshold of 0.4. ‘-i’ signifies that smaller dissimilarity scores were obtained for inverted regulator expression (d^(gR,gT,mΔT)<d(gR,gT,mΔT)∀m); ‘-a’ signifies that smaller dissimilarity scores were obtained for non-inverted regulator expression (d(gR,gT,mΔT)<d^(gR,gT,mΔT)∀m). (TIFF) [file pone.0136591.s001.tiff]

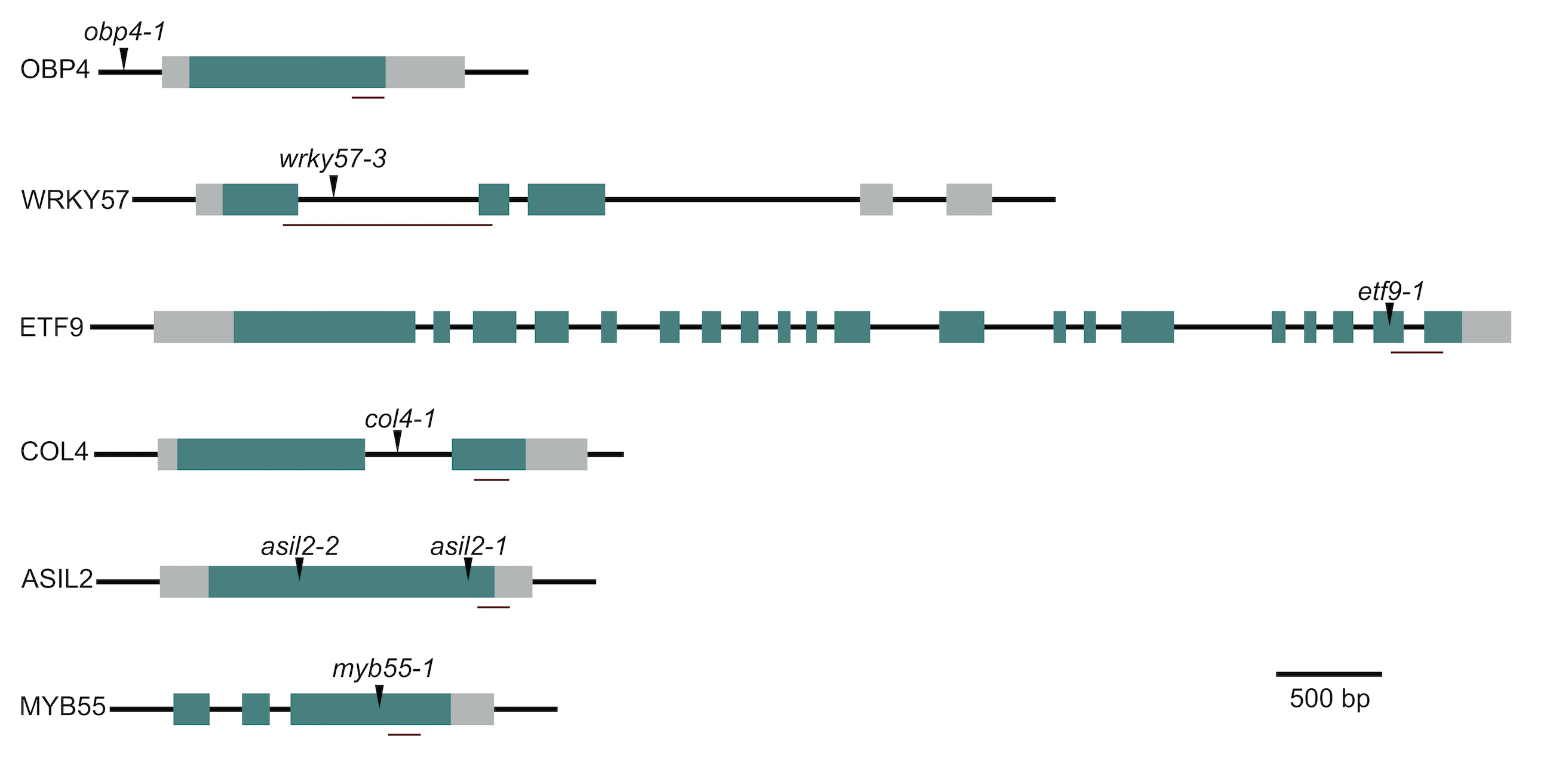

Supplement: S2 Fig — Regulator genes shown with exons in blue, untranslated regions (UTR) in gray, and promoters and introns as lines. Insertion locations are indicated with triangles and lines underneath genes indicate region spanned by qRT-PCR primers. (TIFF) [file pone.0136591.s002.tiff]

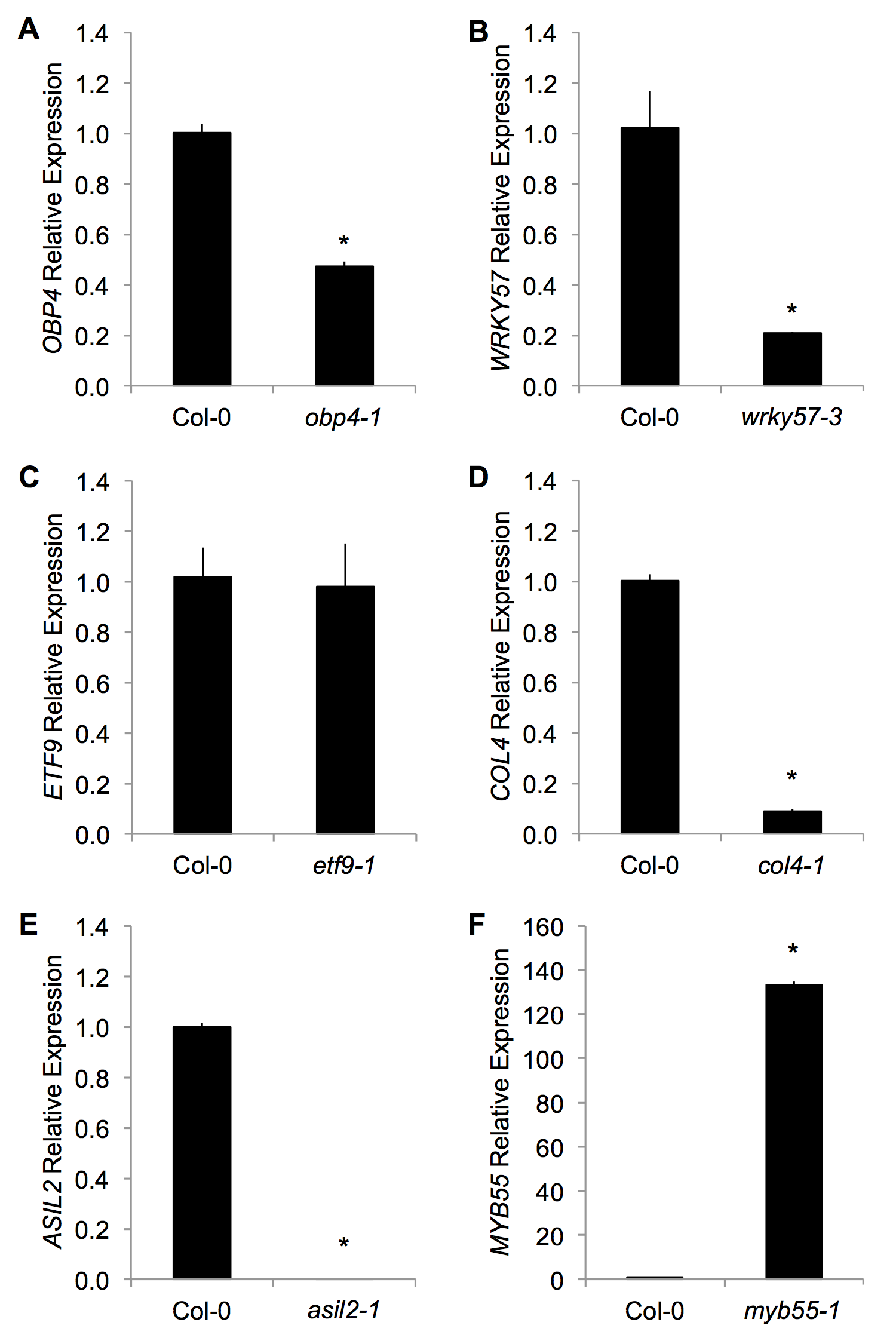

Supplement: S3 Fig — Root tissue was collected from seedlings grown 4 days on iron sufficient media and transferred to iron deficient media for 3 days. Expression values are normalized to β-tubulin and to WT (Col-0) expression for each gene. Error bars indicate ±SEM (n = 4). Expression of (A) OBP4, (B) WRKY57, (C) ETF9, (D) COL4, (E) ASIL2, and (F) MYB55 in respective mutant regulator backgrounds. Asterisk indicates significant difference from WT (Student’s t-test, p < 0.05). (TIFF) [file pone.0136591.s003.tiff]

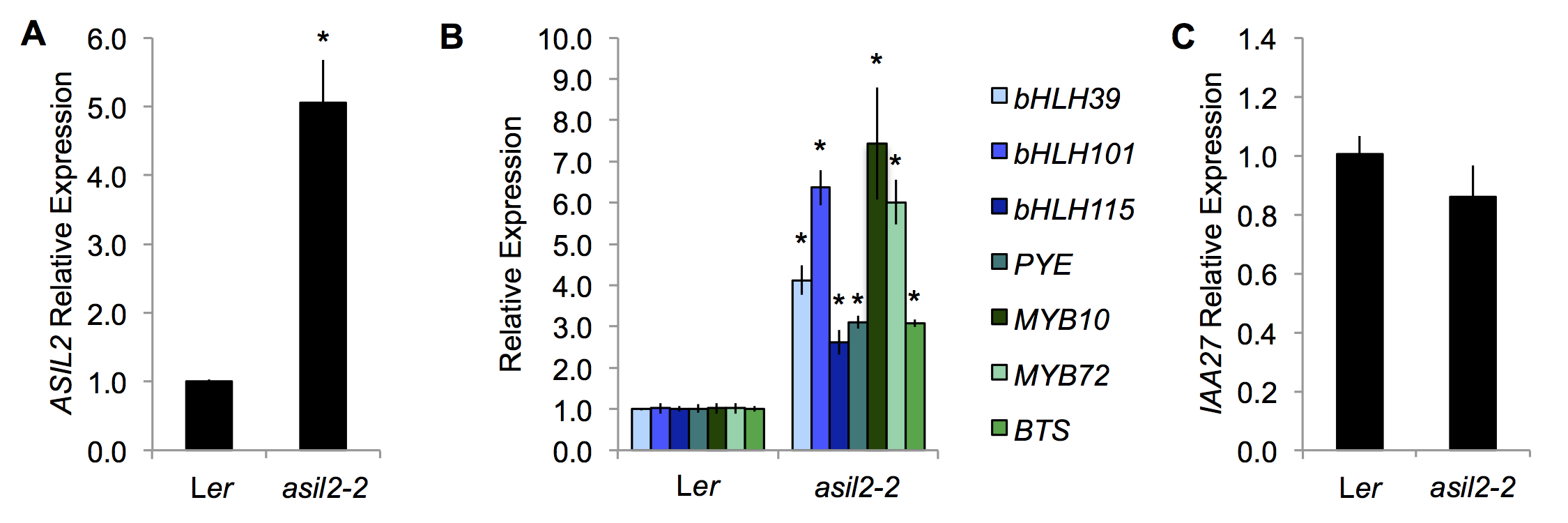

Supplement: S4 Fig — Root tissue was collected from seedlings grown 4 days on iron sufficient media and transferred to iron deficient media for 3 days. Expression values are normalized to β-tubulin and to WT (Ler) expression for each gene. Error bars indicate ±SEM (n = 4). Expression of (A) ASIL2 regulator, (B) ASIL2 targets, and (C) negative control gene IAA27 in asil2-2 mutant background. Asterisk indicates significant difference from WT (Student’s t-test, p < 0.05). (TIFF) [file pone.0136591.s004.tiff]

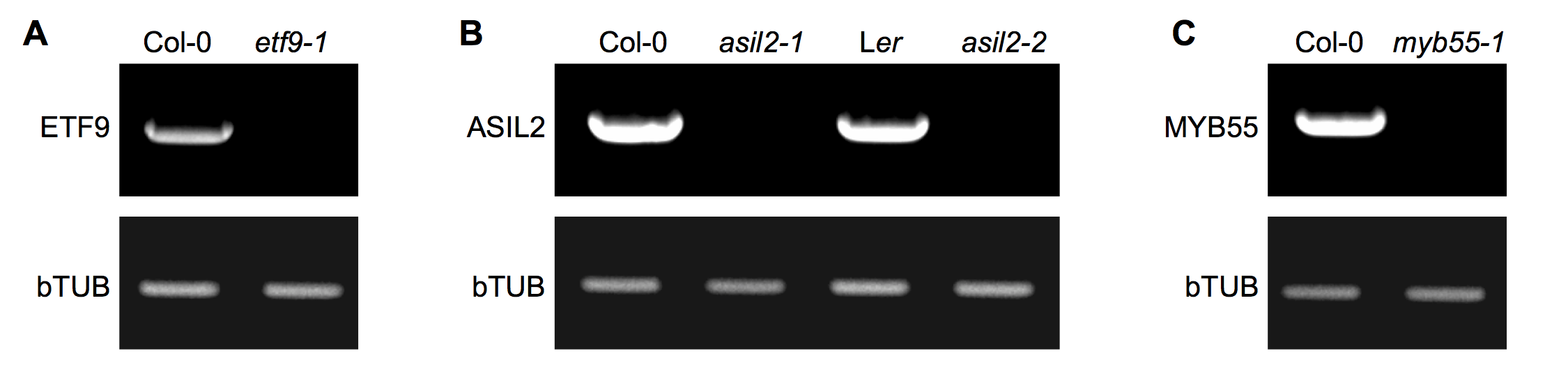

Supplement: S5 Fig — Root tissue was collected from seedlings grown 4 days on iron sufficient media and transferred to iron deficient media for 3 days. PCR was performed on cDNA using primers for full length product (TOPO F&R) for (A) ETF9, (B) ASIL2, and (C) MYB55, each shown with β-tubulin (bTUB) transcript as a control and run until saturation (35 cycles). (TIFF) [file pone.0136591.s005.tiff]

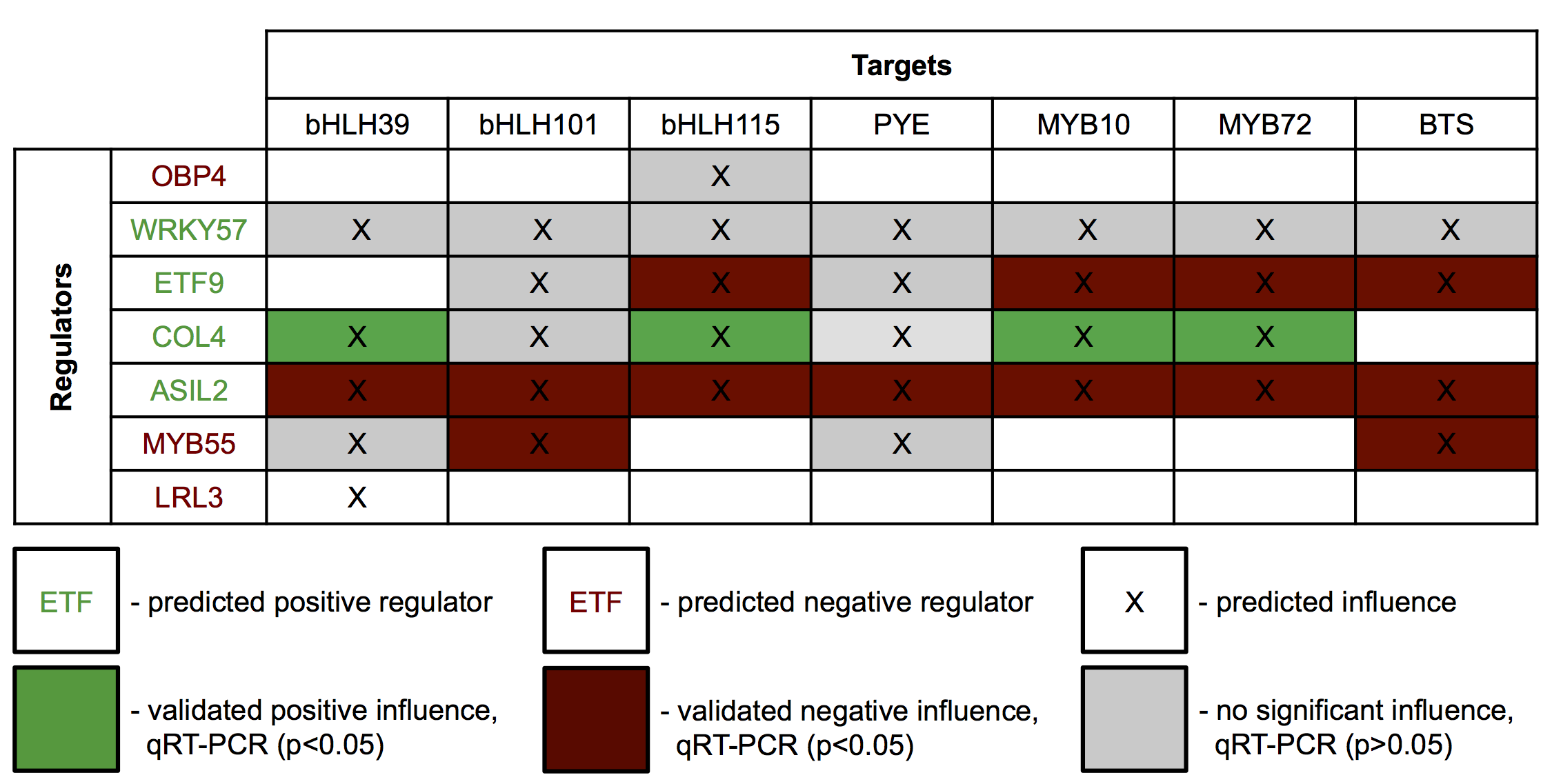

Supplement: S6 Fig — (TIFF) [file pone.0136591.s006.tiff]

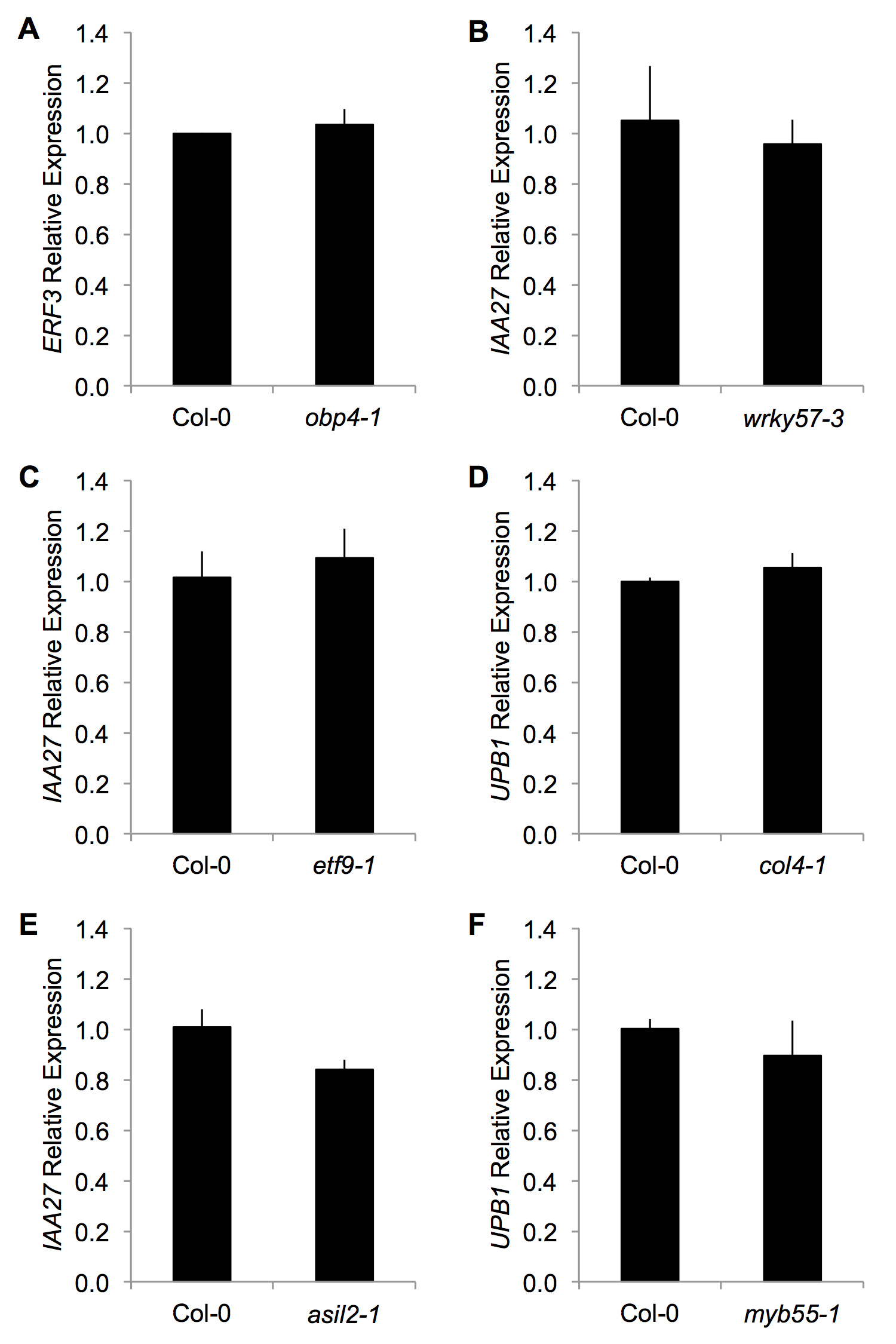

Supplement: S7 Fig — Root tissue was collected from seedlings grown 4 days on iron sufficient media and transferred to iron deficient media for 3 days. Expression values are normalized to β-tubulin and to WT (Col-0) expression for each gene. Error bars indicate ±SEM (n = 4). Expression of (A) ERF3, (B) IAA27, (C) IAA27, (D) UPB1, (E) IAA27, and (F) UPB1 negative control genes in mutant regulator backgrounds. All values are not significantly different from WT (Student’s t-test, p < 0.05). (TIFF) [file pone.0136591.s007.tiff]

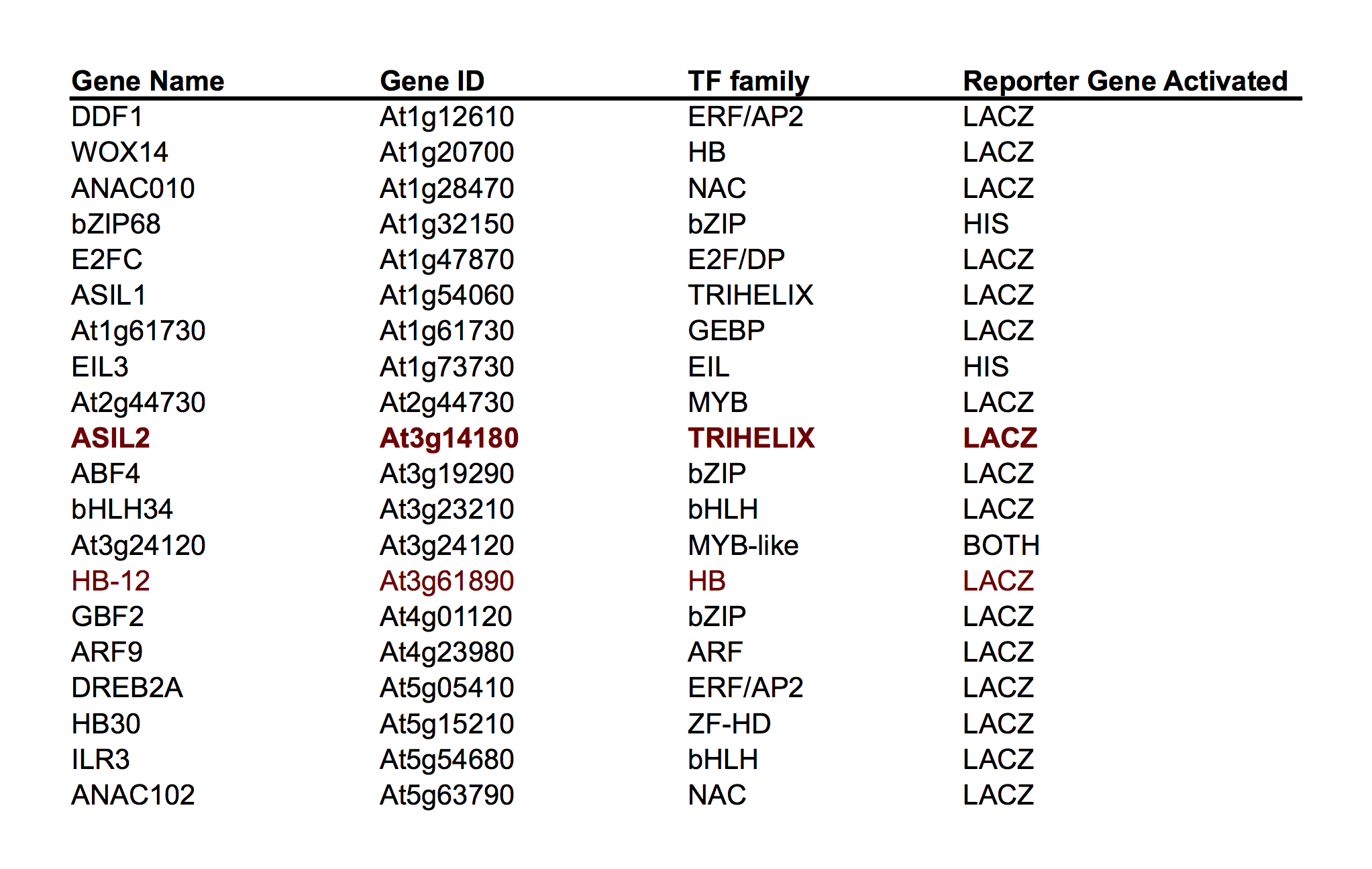

Supplement: S1 Table — Transcription factors with gene activity under iron deficiency are indicated in red and the connection predicted by the CDAA is indicated in bold. (TIFF) [file pone.0136591.s008.tiff]

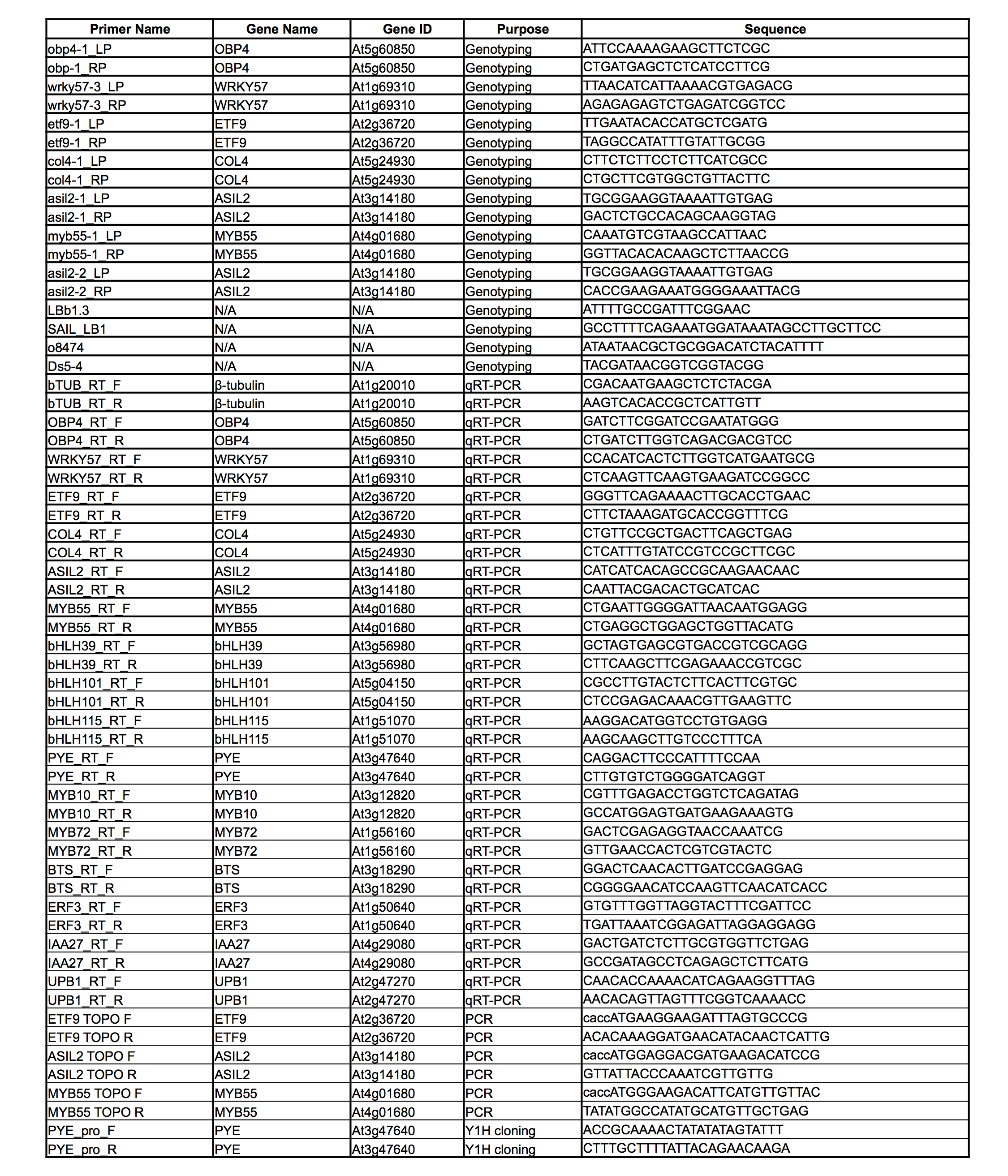

Supplement: S2 Table — (TIFF) [file pone.0136591.s009.tiff]

# Dissimilarity tables

## bHLH115

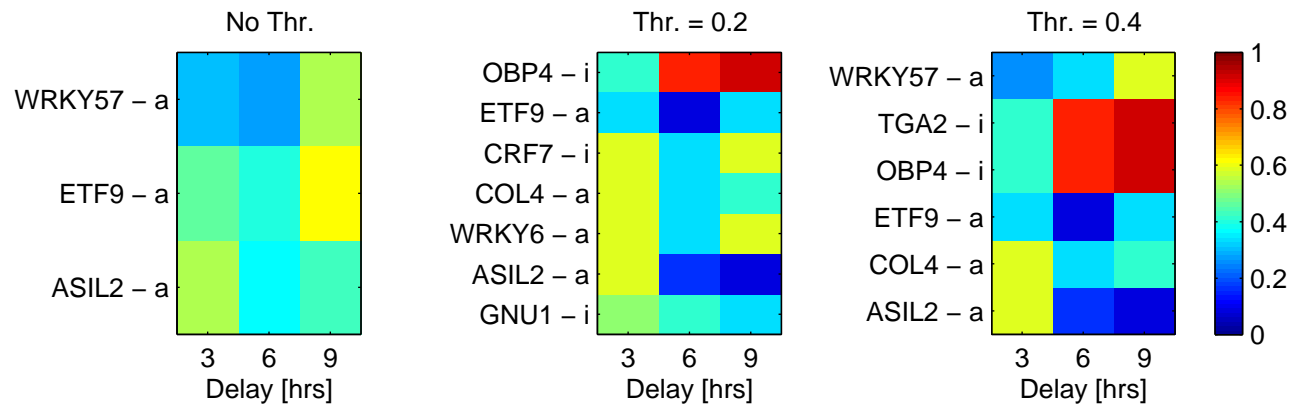

## MYB72

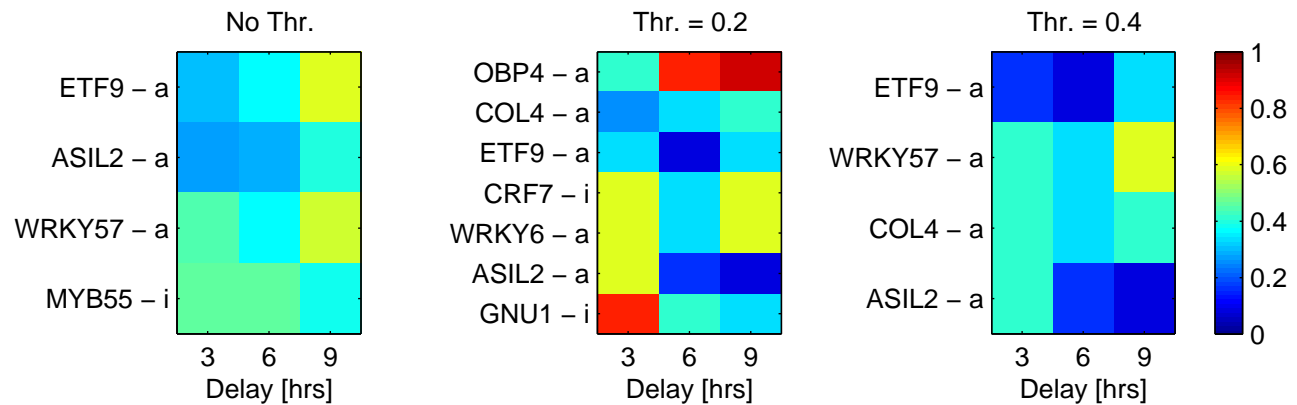

## MYB10

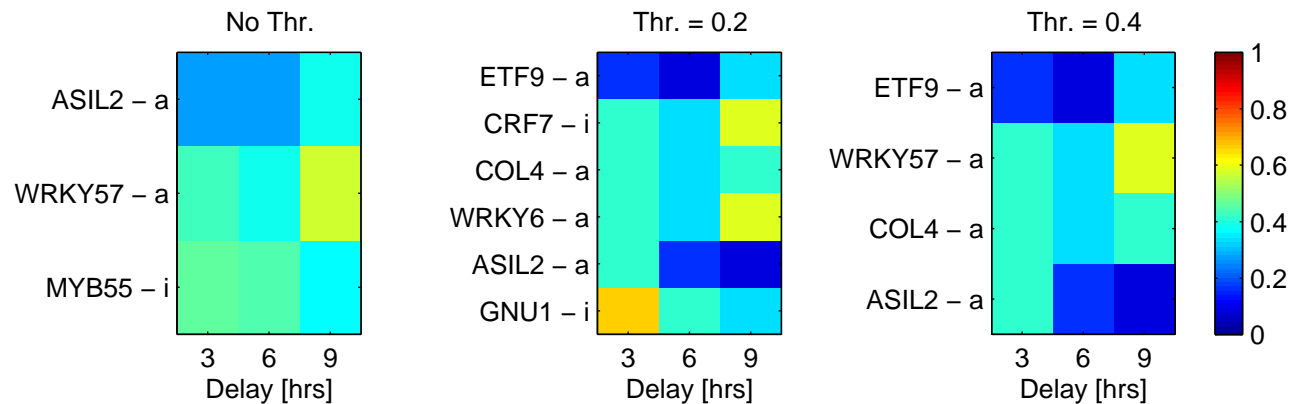

## PYE

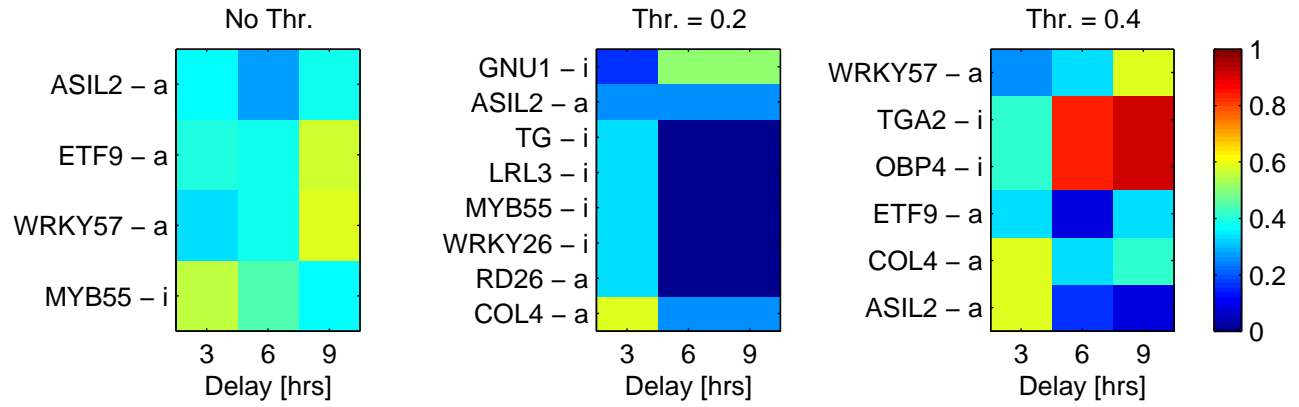

## bHLH101

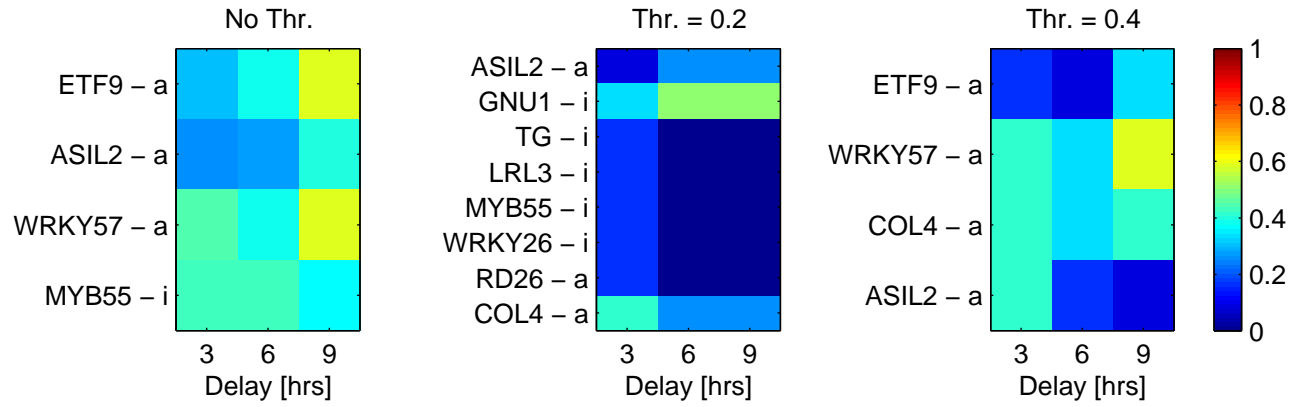

## bHLH39

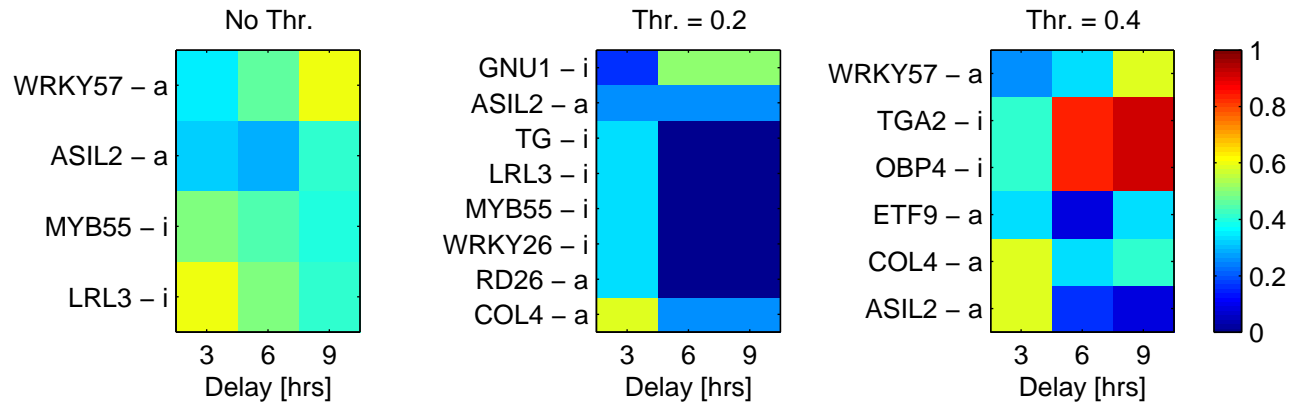

# BTS

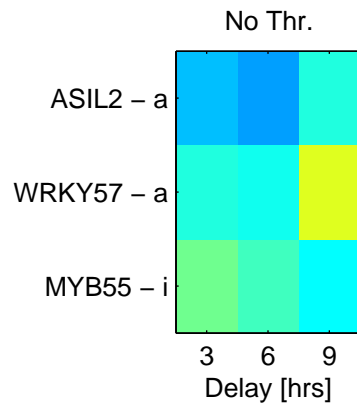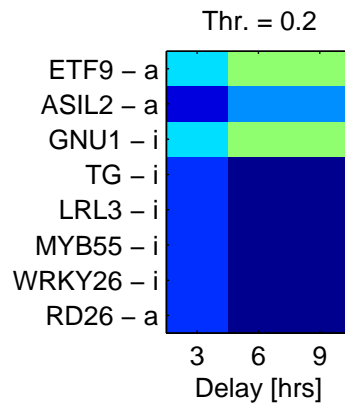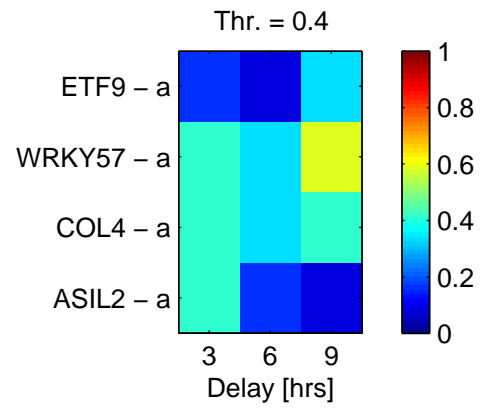

Supplement: S5 File — (PDF) [file pone.0136591.s014.pdf]
